# A Chemical Foundation Model Benchmark for PFAS Bioactivity Prediction: Multi-Task Fine-Tuning Beats Fingerprint Baselines, PFAS Domain Pretraining Does Not Help

**DOI:** 10.1021/acs.jcim.6c02202

**Published:** 2026-09-24

**Authors:** Yue Zhu, Qingyang Liu

**Affiliations:** † College of Computing, 1372 Georgia Institute of Technology, 225 North Avenue NW, Atlanta, Georgia 30332, United States; ‡ Khoury College of Computer Sciences, 1848 Northeastern University, 360 Huntington Avenue, Boston, Massachusetts 02115, United States

## Abstract

Per- and polyfluoroalkyl substances (PFAS) comprise more than 21,000 chemicals, yet fewer than 1.3% have any experimental bioactivity measurement, making structure-based hazard prediction the gating problem for prioritizing the untested majority. Existing PFAS models are single-task and rarely evaluated under leakage-aware cross-validation. We present the first multitask chemical foundation-model benchmark for PFAS bioactivity, fine-tuning off-the-shelf MolFormer-XL with 156 ToxCast classification heads under Tanimoto-Butina cluster-out cross-validation, which is appropriate because 89% of PFAS have empty Murcko scaffolds. The model reaches macro AUROC 0.764, beating a Morgan-fingerprint XGBoost baseline by +0.18 and winning on 155 of 156 end points. That baseline is, however, partly degenerate: binary Morgan fingerprints map a quarter of the compounds onto identical vectors, and sweeping eight 1*D*/2D/3D representations on the same folds raises the best classical model to 0.688, so the margin properly attributable to the foundation model is +0.076. A matched factorial ablation shows that backbone fine-tuning and multitask learning are synergistic: multitask learning helps only once the backbone is fine-tuned (+0.08). This interaction is backbone- and task-dependent: it reproduces on Tox21 for two backbones but is absent for ChemBERTa-2 on PFAS, and label-free encoder diagnostics show this is not because ChemBERTa-2 represents PFAS poorly, so static representation quality does not predict postfine-tuning performance. PFAS-specific domain-adaptation pretraining gives no detectable improvement (Δ = – 0.01 to – 0.03). Mondrian-by-class conformal calibration improves rare-active-class coverage from 0.36 to 0.78, and an applicability-domain analysis shows only 26% of untested PFAS lie close enough to the training set for the model to be applied without caveat. The representation also transfers to soil-water partitioning (*K*_d_) regression, where it holds at Spearman ρ = 0.75 even on PFAS chemistries absent from training while the fingerprint baselines collapse.

## Graphical abstract

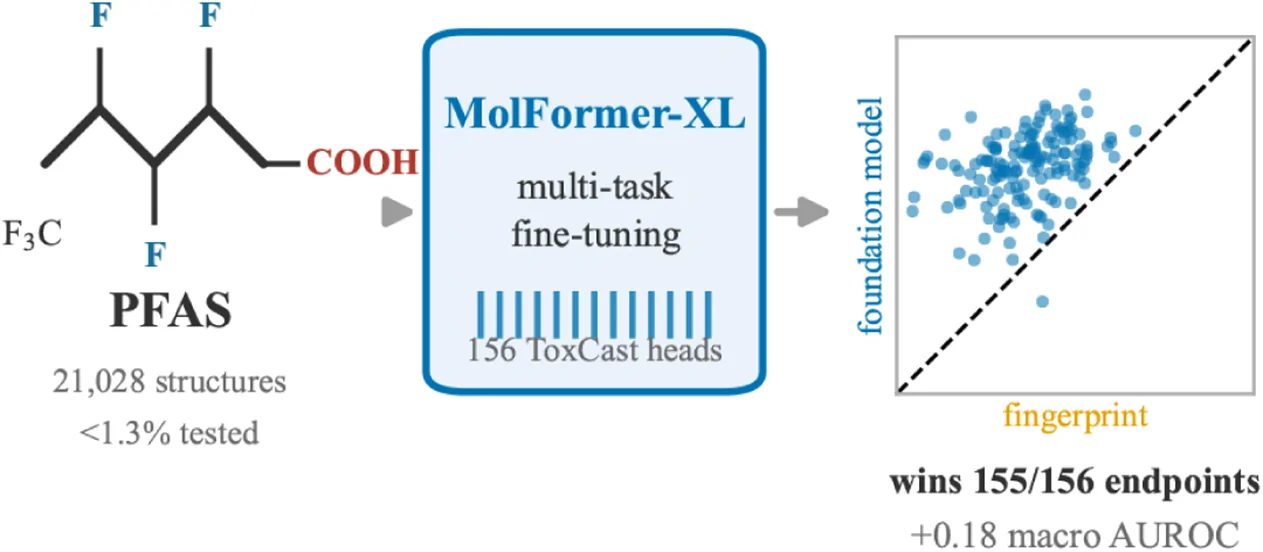

## Introduction

Per- and polyfluoroalkyl substances (PFAS) are a class of more than 21,000 synthetic fluorinated chemicals whose carbon–fluorine bonds confer exceptional thermal and chemical stability.^1^ Widespread industrial use, environmental persistence, and documented associations with immunotoxicity, endocrine disruption, thyroid dysfunction, and hepatotoxicity have made PFAS contamination a global regulatory priority.^2^ Yet experimental hazard data remain extremely sparse: of 21,028 PFAS in the EPA CompTox PFASSTRUCT V6 list, only 275 (1.3%) appear anywhere in the ToxCast invitroDB v4.3 database, and only 156 end points carry enough PFAS-annotated entries to support model evaluation. Computational prediction of PFAS hazard from chemical structure is therefore essential for prioritizing which of the remaining 99% of structures deserve experimental attention first.

Existing machine-learning approaches to PFAS property prediction face two compounding methodological problems that inflate reported performance. First, conventional scaffold-out cross-validation, the cheminformatics standard for avoiding train/test leakage, breaks down for PFAS: 89% of the 21,028 PFAS in PFASSTRUCT V6 are linear perfluoroalkyl chains with no ring system, so their Murcko scaffold is the empty string.^3^ Applying scaffold-out CV to a data set where almost all molecules share the same empty “scaffold” yields a single trivial fold rather than structure-separated splits. Second, single-task QSAR on the small per-end point PFAS slices (typically 100–300 labeled compounds) is unstable under structure-based CV: 86% of Morgan-fingerprint plus random-forest single-task baselines (122 of 142 end points) land in a “degenerate” bin with mean AUROC below 0.50 or a majority of undefined folds under Tanimoto-Butina cluster-out CV, too noisy to rank methods reliably.

Chemical foundation models, large transformers pretrained on hundreds of millions to billions of SMILES strings, offer a natural remedy for data scarcity: multitask fine-tuning over a shared backbone lets all 156 end points jointly inform the representation, which increases the effective sample size.^4^ Joint training across many assays is a long-standing fix for data-scarce end points, established by massively multitask networks^5^ and by multitask deep learning on Tox21.^6^ A natural follow-on question is whether domain-specific pretraining of the backbone on PFAS chemical space would further improve performance: domain adaptation can help for niche chemical subspaces,^7^ but PFAS are predominantly short linear fluoroalkyl chains, a chemistry that generalist models may already represent well. We addressed both questions empirically.

The novelty here is not the backbone. MolFormer-XL is an off-the-shelf, extensively benchmarked model, and we deliberately use it unmodified; multitask toxicity modeling likewise predates us.^5,6^ The knowledge gap this paper closes is a PFAS-specific one. For this chemical class it was not known (i) which of the ingredients that a practitioner might reach for actually pays—a better representation, joint training across end points, or backbone adaptation—once leakage-aware splitting removes the inflation that random-split evaluations introduce; (ii) whether the standard fingerprint baseline is even a valid comparator, given that binary Morgan fingerprints collapse chemically distinct long-chain PFAS onto identical vectors (representation results, below); and (iii) whether PFAS-specific domain-adaptive pretraining, the obvious next step for a niche chemistry, is worth its compute. We answer all three on a single controlled benchmark, and two of the three answers are negative. Our contributions are

(1) **Multitask foundation-model benchmark.** Fine-tuning off-the-shelf MolFormer-XL with 156 simultaneous binary heads on ToxCast PFAS bioactivity achieves macro AUROC 0.764 ± 0.100 under 5-fold Tanimoto-Butina cluster-out CV, beating single-task Morgan-FP plus XGBoost by Δ = +0.18 on **155 of 156 end points (99.4%)**. Because binary Morgan fingerprints are a weak comparator on this chemistry, we also sweep eight 1D, 2D and 3D representations on the same folds; the strongest classical alternative (RDKit 2D descriptors plus XGBoost) reaches 0.688, so the honest margin attributable to the foundation model is +0.076 rather than +0.18 (Table 3). A matched factorial ablation shows that backbone fine-tuning and multitask learning are synergistic. Multitask heads add nothing on a frozen backbone but contribute +0.08 on a fine-tuned one, so neither component alone reproduces the result. The synergy reproduces on Tox21 across two backbones but is backbone-dependent: it is absent for ChemBERTa-2 on PFAS, and label-free encoder diagnostics show this is *not* because ChemBERTa-2 encodes PFAS structure poorly (generalization results, below). We therefore report the scope empirically rather than claiming universality.
(2) **Negative domain-adaptation result.** We test two recipes of PFAS-specific masked-language-model pretraining on PFASSTRUCT V6: an aggressive one (30 epochs, LR 5 × 10^–5^) and a gentle one (5 epochs, LR 10^–5^). Both fail to improve over the off-the-shelf backbone (Δ = – 0.012 and – 0.027; both NS, *p* > 0.40). MolFormer’s tokenizer already covers PFAS lexically: it emits 208 out-of-vocabulary tokens in 1,089,670 tokens (0.019%) over the 11,028 unique PFASSTRUCT SMILES, and none at all on the 209 evaluation PFAS, so there is no vocabulary gap for PFAS pretraining to close.
(3) **Cross-task transfer to a regression end point.** Without ever seeing *K*_d_ labels, MolFormer [CLS] embeddings plus a Ridge head reach Spearman ρ = 0.79 ± 0.21 on the Fabregat-Palau 2025 soil-water *K*_d_ data set (42 PFAS, 1,207 measurements),^8^ versus 0.59 ± 0.23 for Morgan-FP plus XGBoost. A leave-overlap-out test (train on 23 shared PFAS, test on 19 with chemistry unseen in the bioactivity set) holds at ρ = 0.75 while fingerprint baselines collapse, so the representation transfers across both property type and unseen chemistry.
(4) **Methodological corrections and reliability scoping**. We introduce an autofallback rule from Murcko to Tanimoto-Butina cluster CV for linear chemistries and show (as an exploratory analysis) that Mondrian-by-class split-conformal calibration mitigates a failure mode of vanilla conformal prediction on imbalanced PFAS end points, raising rare-active-class coverage from 36% to 78%. We also delimit where the model may be used: only 26% of the 20,793 untested PFASSTRUCT compounds lie within a mean-top-5 Tanimoto of 0.4 of the training set (applicability domain), so screening the untested majority requires an explicit domain check rather than blanket application.

At *N* = 10 training PFAS, the multitask model already reaches macro AUROC 0.574, nearly matching the full-*N* single-task XGBoost baseline (0.584). This data efficiency matters most in the low-data regime that characterizes rare PFAS subfamilies (Figure 2).

## Related Work

### PFAS Bioactivity and Toxicity Prediction

Early QSAR work by Cheng and Ng^2^ classified 3,486 OECD-list PFAS with Morgan fingerprints and random forests under random 10-fold CV, reporting accuracies later recognized as optimiztic given the absence of structure-based splitting. Subsequent single-end point models targeted specific interactions: gradient-boosted trees with SHAP for PFAS–PPARα binding,^9^ QSAR for human transthyretin disruption^10^ and its rat cross-species extension,^11^ graph convolutional networks for liver fatty acid-binding protein,^12^ and random forests for toxicokinetic half-lives.^13^ Two studies are closest to ours. Feinstein et al.^14^ (AI4PFAS) transfer-learned a single-end point acute-toxicity model wrapped in a SelectiveNet abstention layer; this combines transfer learning with selective prediction, but covers one end point, uses no foundation-model backbone, and is not evaluated under cluster-out CV. Kwon et al.^15^ used semisupervised metric learning to predict bioactivities across many human targets for the OECD list. This is the nearest multitarget prior art, but it is not a fine-tuned foundation model and does not use leakage-aware splitting. To our knowledge this is the first work to fine-tune a chemical foundation model with shared multitask heads across 156 PFAS ToxCast end points under Tanimoto-Butina cluster-out CV; we scope the novelty claim to this specific combination, not to multitask toxicity modeling in general, which predates us.^5,6^

### PFAS Occurrence and Environmental Fate Prediction

A parallel literature applies ML to PFAS occurrence rather than biological end points: gradient-boosted trees for national groundwater rasters,^16^ multilabel semisupervised learning for 35 PFAS in California groundwater,^17^ interpretable ML for hazard in European surface waters,^18^ ML plus simulation for membrane rejection,^19^ and explainable models for soil-water partitioning (*K*_d_).^8,20^ These works uniformly train separate models per PFAS species or analyte, without exploiting shared chemical structure across the class. A related line of work applies machine learning directly to SMILES-derived representations of aqueous contaminants: Raza et al.^21^ predicted PFAS defluorination from molecular descriptors and fingerprints, and Andronov et al.^22^ showed that difference fingerprints combined with parametric t-SNE organize chemical space in a way that supports downstream analysis. Both illustrate that a well-chosen SMILES-derived representation plus an unsupervised or shallow learner is often competitive; our chemical-space analysis (chemical-space analysis, below) uses t-SNE in the same spirit, and our representation sweep (Table 3) quantifies how far that route goes on this task.

### Chemical Foundation Models

Large-scale SMILES pretraining yields general-purpose encoders for few-shot transfer. Ross et al.^4^ trained MolFormer-XL on 1.1 billion ZINC/PubChem SMILES with linear attention, reaching state-of-the-art on MoleculeNet^23^ without fine-tuning; ChemBERTa-2 ^7^ adapted RoBERTa to 10 million PubChem SMILES. Whether a *further* round of domain-adaptive pretraining helps is unsettled: in NLP, continued in-domain pretraining reliably helps,^24^ but in the molecular domain Sun et al.^25^ found self-supervised GNN pretraining often gives no significant gain over training from scratch. Our negative Stage-1 result is consistent with the latter, on a SMILES foundation model. Multitask learning as a remedy for label scarcity predates these models^5,6^ and remains an active strategy for low-data toxicological end points, where Wichrowski et al.^26^ also document the structured missingness that assay-panel design imposes on such label matrices—a confound we quantify for our own data in the missingness analysis below. Graph approaches such as message-passing networks^27^ and domain-specific architectures like MolHGT+^28^ have been applied to PFAS. Graph models support multitask training perfectly well—multitarget heads are a standard feature of Chemprop,^29^ and Javaid et al.^30^ study multitask graph learning for molecular property prediction directly. What distinguishes the setting here is not the architecture’s ability to carry many heads but the starting point: graph models for PFAS are typically trained from scratch on the target labels, whereas the question we ask is whether a *pretrained* SMILES encoder supplies the transferable chemistry. Our from-scratch multitask message-passing baseline (B4, main comparison) isolates exactly that distinction.

### Conformal Prediction and Chemical Cross-Validation

Conformal prediction^31,32^ gives distribution-free coverage via prediction sets; Mondrian conformal prediction^33^ extends coverage to conditional subgroups (here active vs inactive), critical for imbalanced data where vanilla conformal under-covers the rare class. It has been introduced to QSAR as a transparent alternative to applicability-domain estimation,^34^ but its use in multitask molecular models remains limited. On splitting, scaffold-out CV^3^ is the standard against train/test leakage; Yang et al.^35^ showed it both lowers and reorders reported performance relative to random splits, and Roberts et al.^36^ demonstrated analogous cluster-out principles for spatially structured data. Butina^37^ introduced the Tanimoto clustering underlying our fallback, and Yang et al.^38^ cataloged CV pitfalls in environmental ML. We extend these contributions by specifying the threshold (fewer than 30 unique nonempty Murcko scaffolds) at which scaffold CV should automatically fall back to Tanimoto-Butina clustering, a rule that applies to any linear-chain chemistry and not only to PFAS.

## Methods

### Data

#### Bioactivity Labels

We obtained the ToxCast invitroDB v4.3 multiconcentration database from the EPA CompTox dashboard.^39,40^ Of the 21,028 PFAS in the EPA PFASSTRUCT V6 list^1^ (each with a DTXSID and SMILES), 275 have been screened in invitroDB v4.3 and 209 carry a usable binary hitcall. We select evaluable end points by two filters: ≥ 100 distinct PFAS labeled per end point, and ≥ 10 active hits per end point. This removes 120 constant-label end points (including the entire ATG_CIS panel, where no tested PFAS is active) and yields **156 ToxCast end points** spanning 13 intended target families (invitroDB v4.3 annotation). The surviving panel is dominated by cell-based phenotypic readouts: 71 cytokine end points, 31 cell-adhesion-molecule end points, 15 protease, 12 cell-morphology, 7 cell-cycle and 7 zebrafish-development end points, with GPCR, kinase and nuclear-receptor targets represented by only 4, 3, and 1 end points respectively—the transcription-factor panels that would have supplied most nuclear-receptor end points are exactly the ones removed by the ≥ 10-active filter, because essentially no tested PFAS is active in them. SI Tables S12 and S13 give the per-end point catalog and the roll-up by target family. Each end point is labeled for exactly 168 PFAS; the union across all 156 end points is **209 distinct PFAS**. A given end point therefore lacks labels for 41 of the 209-compound union, and the binary label mask *m*_*e*,*c*_ in eq 1 excludes those (compound, end point) pairs from the loss. The cross-validation folds partition the 209-PFAS union, so per-fold compound counts sum to 209. Table 1 reconciles every count used in this paper.

**Table 1:** Dataset Accounting^a^ ^,^ ^b^

Quantity	Count
PFAS in EPA PFASSTRUCT V6 (source list)	21,028
screened in ToxCast invitroDB v4.3	275
with a usable binary hitcall (mc matrix)	209
ToxCast end points after filtering (≥100 PFAS, ≥ 10 actives)	156
Labeled PFAS per end point	168
Distinct PFAS across all 156 end points (CV union)	209
Mean training-fold size (few-shot *N* = all)	167
End points evaluable by the balanced RF baseline	142

a Every PFAS/end Point Count Used in This Paper, Traced from the Source Chemical List to the Cross-Validation Splits.

b The 5 cluster-out folds hold 42, 42, 42, 42, and 41 PFAS and partition the 209-compound union. RF is evaluable on 142/156 end points because a class-balanced random forest cannot be fit when a training fold of an end point is single-class; XGBoost and the foundation-model variants handle all 156.

This missingness is not random. ToxCast screening of PFAS is organized around the EPA PFAS inventory, so the label matrix is strongly bimodal: 146 of the 209 compounds are screened on all 156 end points while 41 are screened on fewer than ten, giving an overall fill rate of 79.2%. Structured missingness of this kind is a known confound for multitask claims^26^—compounds that appear on many end points could drive an apparent multitask benefit through shared label coverage rather than shared biology—so the missingness analysis below quantifies the structure and tests whether it explains our result.

For stage-1 domain-adaptation experiments, the PFASSTRUCT V6 SMILES serve as the pretraining corpus; after canonicalization and deduplication, the 21,028 structures reduce to **11,028 unique SMILES**.

#### Cross-Task Regression Data Set

To test whether the representation transfers across property types, we use the Fabregat-Palau 2025 soil-water partitioning data set (*K*_d_, log scale) from the Supporting Information of ref 8: 42 PFAS spanning seven subfamilies, with 1,207 (PFAS, soil) measurement pairs averaged to one per-PFAS log *K*_d_ value (averaging over soils makes this a per-PFAS regression on the same inputs and Butina folds as bioactivity). Of the 42 PFAS, 23 share a structure (matching DTXSID or canonical SMILES) with the 209 ToxCast PFAS and 19 do not. The evaluation is first a *cross-task transfer* test (a property type the backbone never saw labeled); to additionally probe unseen chemistry we run a *leave-overlap-out* split that trains on the 23 in-distribution PFAS and tests on the 19 (Table 8; full lists in Table S2). The protocol is identical across representations: one fixed feature vector per PFAS (off-the-shelf MolFormer [CLS] embedding, or 2048-bit Morgan fingerprint) feeding a linear head (RidgeCV with α ∈ {0.1, 1, 10} for MolFormer; the same XGBoost and RF regressors for the baselines). The backbone is never exposed to *K*_d_ labels and is not fine-tuned; only the head is refit on each of the 5 folds. We report Spearman ρ, *R* ^2^, and MAE as mean ± SD over folds.

### Model Architecture

#### Backbone

We use the public ibm/MoLFormer-XL-both-10pct checkpoint^4^ as a frozen or fine-tuned encoder: a 12-layer linear-attention transformer (47 M parameters) with a byte-pair-encoding tokenizer trained on 1.1 billion ZINC/PubChem SMILES. The vocabulary contains 2,362 tokens; tokenizing all 21,028 PFASSTRUCT SMILES produces only 208 unknown tokens out of 1,089,670 (0.019%), confirming near-complete coverage of perfluoroalkyl chemistry.

#### Multitask Classification Heads

On the [CLS] vector we place a shared 768 → 512 projection (GELU, dropout 0.1) feeding 156 independent linear heads, one per end point (Figure 1; *f*_*e*_ denotes the end point-*e* head). Training uses masked binary cross-entropy:

1 $$L_{\mathrm{BCE}}=-\frac{1}{\left|E\right|}\underset{e\in E}{\sum }\frac{1}{\underset{c}{\sum }m_{e,c}}\underset{c}{\sum }m_{e,c}\left[y_{e,c}\log{ŷ}_{e,c}+\left(1-y_{e,c}\right)\log\left(1-{ŷ}_{e,c}\right)\right]$$

where *y*_*e*,*c*_ ∈ {0, 1} is the label, *ŷ*_*e*,*c*_ = σ­(*f*_*e*_([CLS]_*c*_)) the predicted probability, and *m*_*e*,*c*_ ∈ {0, 1} the label mask. The mask lets compounds with incomplete coverage contribute without imputing missing labels. Training runs 5 epochs with the backbone frozen (head warmup) then 45 epochs of full fine-tuning at a cosine-annealed LR of 3 × 10^–5^, batch size 16, bfloat16, on one RTX 5090 (≈3 min per 5-fold run).

**Figure 1:**
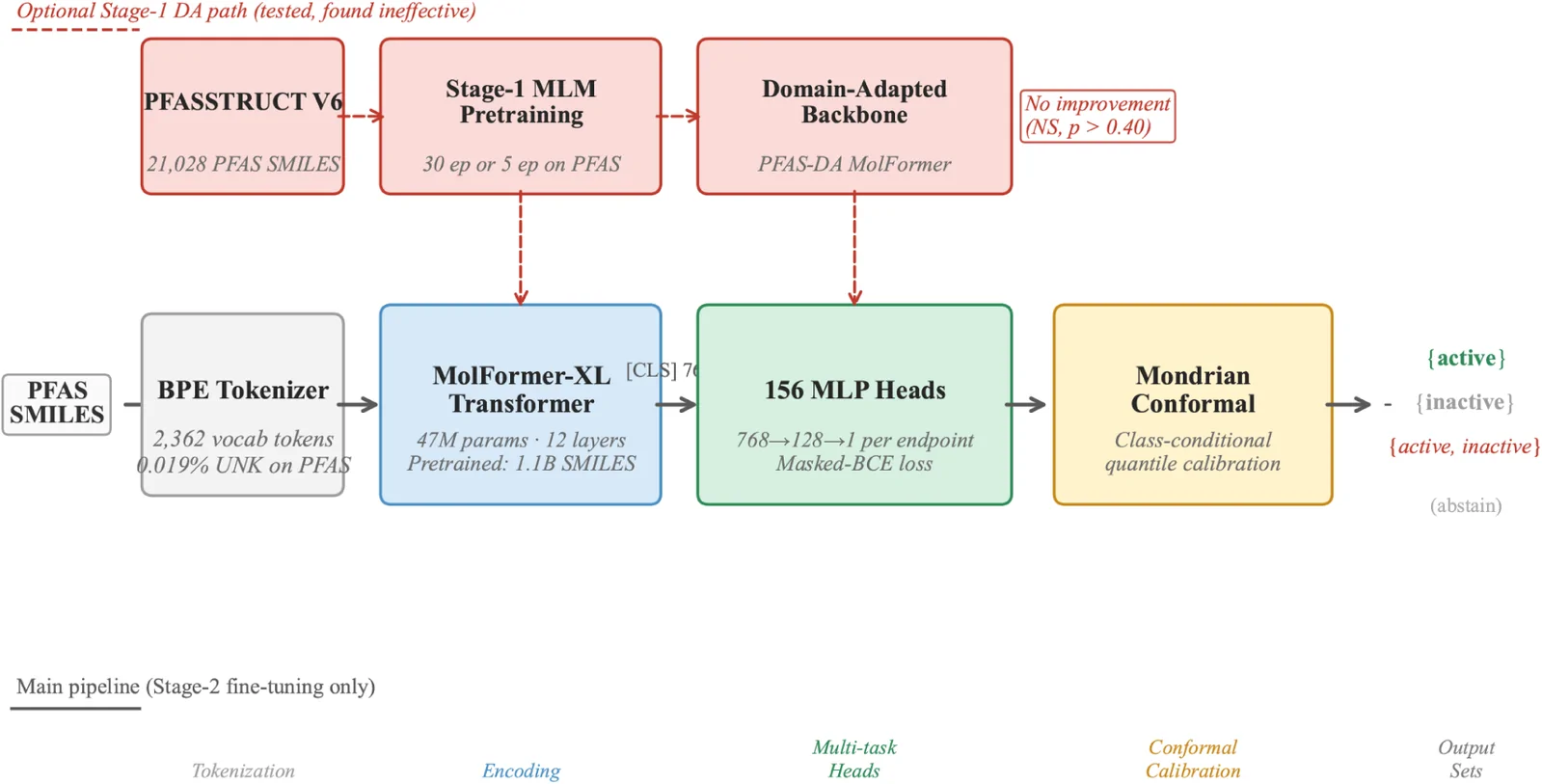
Pipeline overview. A frozen or fine-tuned MolFormer-XL encoder maps PFAS SMILES to a 768-dimensional [CLS] representation feeding 156 independent multitask classification heads trained with masked binary cross-entropy. The optional Stage-1 domain-adaptation block (dashed) is evaluated as an ablation.

#### Stage-1 Domain Adaptation

For the ablation we prepend a masked-language-model pretraining stage on PFASSTRUCT V6 SMILES, testing two recipes spanning a 6 × range in LR × epoch space: aggressive (30 epochs, LR 5 × 10^–5^) and gentle (5 epochs, LR 10^–5^, controlling for overtraining).

### Cross-Validation Protocol

Murcko scaffold-out CV requires enough distinct nonempty scaffolds to form meaningful splits; we adopt 30 as a practical trigger. For PFAS this fails: of the 209 PFAS in our benchmark, 187 (89.5%) have an empty Murcko scaffold and the remaining 22 span just **15 distinct scaffolds**, well below 30. A standard scaffold split therefore either places all 187 acyclic PFAS in one group or splits them arbitrarily by SMILES order, providing no structural separation. We therefore use **5-fold Tanimoto-Butina cluster-out CV** (similarity cutoff 0.6)^37^ as an automatic fallback, implemented in our open-source ScaffoldKFold: if fewer than 30 distinct nonempty Murcko scaffolds exist, the splitter falls back to ButinaKFold. The 30-scaffold trigger and the 0.6 cutoff are community-standard heuristics rather than tuned optimal values; varying the cutoff over {0.5, 0.6, 0.7, 0.8} leaves the partition essentially unchanged (41–42 compounds per fold throughout; Table S5). The 5 folds at cutoff 0.6 contain 42, 42, 42, 42, and 41 PFAS (sum 209). All models, both baselines and foundation-model variants, are trained and evaluated on these same folds with matched seeds.

#### Robustness of the Partition

Two checks probe whether conclusions depend on the particular partition. First, the whole 5-fold protocol is repeated under four additional split seeds, each of which regenerates the Butina partition and retrains the headline model from scratch (repeated partition results, below). Second, the leave-one-subfamily out analysis of the subfamily results removes an entire chemical subfamily at a time. We did *not* run leave-one-out CV at the level of individual clusters: the 209 compounds form 108 Butina clusters of which most are singletons, so per-cluster AUROC is undefined, and a pooled variant would answer a different question than the fold-level protocol.

### Baselines

**B1: Morgan-FP + RF.**2048-bit Morgan fingerprints (radius 2); one scikit-learn random forest per end point (500 trees, balanced class weight, defaults otherwise). 142 of 156 end points are evaluable (others have single-class test folds yielding undefined AUROC, dropped from that end point’s mean).

**B2: Morgan-FP + XGBoost**.Same fingerprints; one XGBoost classifier per end point^41^ (500 trees, depth 6, LR 0.05, histogram method, scale_pos_weight set per fold for class balance), all 156 end points.

**B3: Off-the-shelf MolFormer (no DA).**MolFormer-XL with no Stage-1 pretraining; identical 156-head architecture and Stage-2 training as the main model. This is the primary comparison for domain adaptation ablation.

**B4: Multitask graph neural network.**A GINE-style message-passing network^42^ trained from scratch on RDKit atom/bond features (4 layers, width 128, mean+max pooling) with the *same* 156-end point shared head and masked-BCE objective (200 epochs, AdamW, LR 10^–3^, same 5 folds). A graph-representation comparator with no large-scale pretraining; pure-PyTorch scatter, no external graph library.

**B5: Morgan-FP + multitask MLP.**The same 2048-bit fingerprints fed to a *multitask* MLP with the identical head architecture, optimizer and epoch budget as the frozen cells of the factorial ablation (2048 → 512 GELU trunk, 156 outputs, masked BCE, 200 epochs, AdamW at LR 10^–3^). This isolates the multitask paradigm from the foundation-model representation: it applies joint training to the fingerprint baseline, so any remaining gap is attributable to the representation rather than to the paradigm.

#### Why These Baselines

The four comparators are chosen to bracket the design space along the axes the paper varies, rather than to survey QSAR practice. B1 (random forest) is the representation-plus-model combination used in the earliest PFAS QSAR work^2^ and remains the field’s default, so it fixes the historical reference point. B2 (XGBoost) is the strongest single-task learner on the same features and is the primary headline comparator, so the foundation model is not compared against a deliberately weak tree. B4 (graph network) supplies a learned, nonfingerprint representation trained *from scratch*, which separates “a learned molecular representation helps” from “a *pretrained* representation helps”. B5 supplies the multitask paradigm on fingerprint features, separating “joint training helps” from “the backbone helps”. Both tree baselines use fixed hyperparameters with *no* per-end point tuning, matching the no-tuning protocol applied to the foundation-model variants, so the comparison reflects out-of-the-box behavior.

#### Representation Sweep

Because binary Morgan fingerprints turn out to be a degenerate representation for long-chain PFAS (representation results, below), we additionally sweep eight molecular representations spanning 1D, 2D, and 3D descriptor families on the *same* five folds: a 12-feature 1D composition vector (molecular weight, carbon and fluorine counts, F/C ratio, longest perfluoroalkyl chain, heteroatom counts, heavy atoms, rotatable bonds, rings); binary Morgan at radius 2 and 3; count-based Morgan at radius 2; 167 MACCS keys; the full RDKit 2D descriptor block; RDKit 2D descriptors concatenated with count Morgan; and a 3D block of RDKit shape descriptors plus AUTOCORR3D computed on an ETKDGv3 conformer optimized with MMFF. Each representation is run through both the B2 (single-task XGBoost) and B5 (multitask MLP) protocols unchanged. Constant columns are dropped and features are standardized on the training fold only.

#### Factorial Ablation

Relative to a fingerprint baseline, the headline comparison varies three factors together: the molecular representation, whether the backbone is fine-tuned, and the learning paradigm. To isolate the two factors internal to the foundation-model pipeline we run a matched 2 × 2 ablation: MolFormer-XL with the backbone ∈ {frozen, fine-tuned} crossed with ∈ {single-task, multitask} heads. All four cells share one head (the 768 → 512 GELU trunk), optimizer, 50-epoch budget, batch size 16, cosine LR 3 × 10^–5^, and the 5 Butina folds; frozen cells hold the backbone fixed, fine-tuned cells unfreeze it after 5 warm-up epochs. Within each paradigm the cells differ only in whether the backbone updates, and within each backbone state only in head count. The single-task cells fit one model per end point (780 fits over 5 folds per backbone state); the multitask cells fit one 156-head model under eq 1, the fine-tuned multitask cell being the headline model. Frozen cells are trained on [CLS] embeddings extracted once from the off-the-shelf backbone. To test how far the interaction extends, we rerun the same four-cell design on three MoleculeNet benchmarks (Tox21, SIDER, ClinTox; scaffold 3-fold CV) and with a second backbone, ChemBERTa-2 (DeepChem/ChemBERTa-77M-MLM), all cells at the full headline budget; the MoleculeNet runs use 3 folds with no per-fold significance test and remain a qualitative external check. Full design and per-fold values are in Table 7.

#### Reporting Conventions

“Macro AUROC” is the mean per-end point AUROC within a fold, reported as mean ± SD across the 5 folds. The per-end point comparisons in the multitask comparison (paired *t*, Wilcoxon) treat the 156 end points as paired observations; because end points share compounds and overlapping pathways they are not independent, so these *p*-values are descriptive effect-strength indicators, not strict tests. The domain-adaptation tests (Table 4) instead use the 5 folds as paired observations (*n* = 5).

#### Threshold-Dependent Metrics

AUROC and AUPRC are threshold-free, but the active class is rare (mean active rate 0.208 across the 156 end points, range 0.044–0.550), so we also report F1, Matthews correlation coefficient (MCC) and balanced accuracy. Since these depend on an operating point, the rule is stated explicitly: for each end point and each fold, the probability cut is the one maximizing F1 on the *training* fold only, never using held-out data. A fixed 0.5 cut is reported alongside it.

#### Applicability Domain

For each held-out compound we define the distance to the model’s domain as the mean Tanimoto similarity (ECFP4, 2048 bit) to its five nearest training-fold compounds, computed under the same 5-fold cluster-out split. Held-out compounds are binned into quartiles of this quantity and per-end point AUROC is recomputed within each bin, which asks directly whether accuracy decays away from the training set. The same measure is then applied to the 20,793 PFASSTRUCT compounds outside the evaluation set to quantify how much of the untested PFAS universe the model may legitimately be applied to.

#### Label-Free Encoder Diagnostics

To characterize a backbone as a PFAS encoder without reference to any downstream score, we compute, on frozen mean-pooled embeddings of the 209 PFAS: the out-of-vocabulary token rate and tokens per heavy atom; the mean pairwise cosine similarity and the effective rank of the embedding covariance (its participation ratio ${\left(\sum_{i}\lambda_{i}\right)}^{2}/\underset{i}{\sum }\lambda_{i}^{2}$ ), which together measure representational collapse; the Spearman correlation between embedding distance and Tanimoto distance; the cross-validated *R* ^2^ of a ridge probe predicting perfluoroalkyl chain length; and 1-nearest-neighbor subfamily retrieval accuracy. A set of 1,000 drug-like Tox21 compounds serves as a reference.

### Conformal Calibration

We apply Mondrian-by-class split-conformal calibration^32,33^ as a posthoc layer on the off-the-shelf multitask model (MAPIE^43^), with *no* model refitting: a single frozen Stage-2 checkpoint produces all probabilities, and calibration only estimates per-class score quantiles. For each end point we compute nonconformity scores (1 – *ŷ*_*e*,*c*_ for *y* = 1, *ŷ*_*e*,*c*_ for *y* = 0) on a calibration set and estimate class-conditional quantiles *q̂*_+_, *q̂*_–_ at target 1 – α = 0.90; the prediction set is $C_{\alpha }\left(x_{c}\right)=\left\{y\in \left\{0,1\right\}|s\left(x_{c},y\right)\leq {\mathrm{q̂}}_{y}\right\}$ , where a singleton signals confidence and a two-element set signals honest abstention. We report three protocols (full specification in Section S6): a *recycled-fold* protocol whose larger calibration set yields genuinely data-driven quantiles (the substantive result); an exchangeability-respecting *true-holdout* protocol repeated over 200 splits per fold, which at *n*_cal_ ≈ 20 is dominated by a conservative small-sample fallback (∼74% of end points); and a *pooled* protocol over all 5-fold-models’ out-of-fold predictions (∼83 calibration compounds, 1% fallback) that escapes that regime at the cost of approximate exchangeability. Empty prediction sets are resolved to the arg-max class. Given the small calibration sizes we present conformal calibration as an exploratory protocol rather than a coverage-guaranteed result.

### PFAS Subfamily Classification

PFAS were classified into subfamilies by SMILES pattern matching: perfluorocarboxylic acids (PFCA), perfluorosulfonic acids (PFSA), perfluoroether carboxylic acids (PFECA, including GenX/HFPO–DA), fluorotelomer alcohols (FTOH), perfluoroalkyl sulfonamides (FOSA/FOSE), and unknown/complex substances (UVCB_PROFLUO). This classifier is used only for per-subfamily analysis (subfamily results, below) and not for any training or CV split.

## Results

### Multi-Task Fine-Tuning Outperforms Single-Task Fingerprint Baselines

Off-the-shelf MolFormer-XL with 156 multitask heads achieves macro AUROC 0.764 ± 0.100 across all 156 ToxCast end points, versus 0.584 ± 0.080 for Morgan-FP plus XGBoost (+0.18) and 0.40 for Morgan-FP plus RF on its 142 evaluable end points (+0.36); the from-scratch multitask GNN (B4) reaches 0.655, above the fingerprint baselines but well short of the fine-tuned foundation model, so the gain is from pretraining+fine-tuning rather than graph structure alone (Table 2). The multitask model wins on 155 of 156 end points (99.4%; Figure 2A). We treat this gap as an effect size: bootstrapping the 156 per-end point ΔAUROC values gives a 95% CI of [+0.166, + 0.192] (10,000 resamples), so the margin is robustly positive; the end point-level paired *t* and Wilcoxon statistics are reported only as descriptive indicators because the end points are not independent (Methods, “Reporting conventions”). Macro AUPRC, which is prevalence-aware and better suited to imbalanced screening, gives the same result (0.530 ± 0.170 vs 0.361 ± 0.103, + 0.17), so the advantage is not an AUROC artifact.

**Figure 2:**
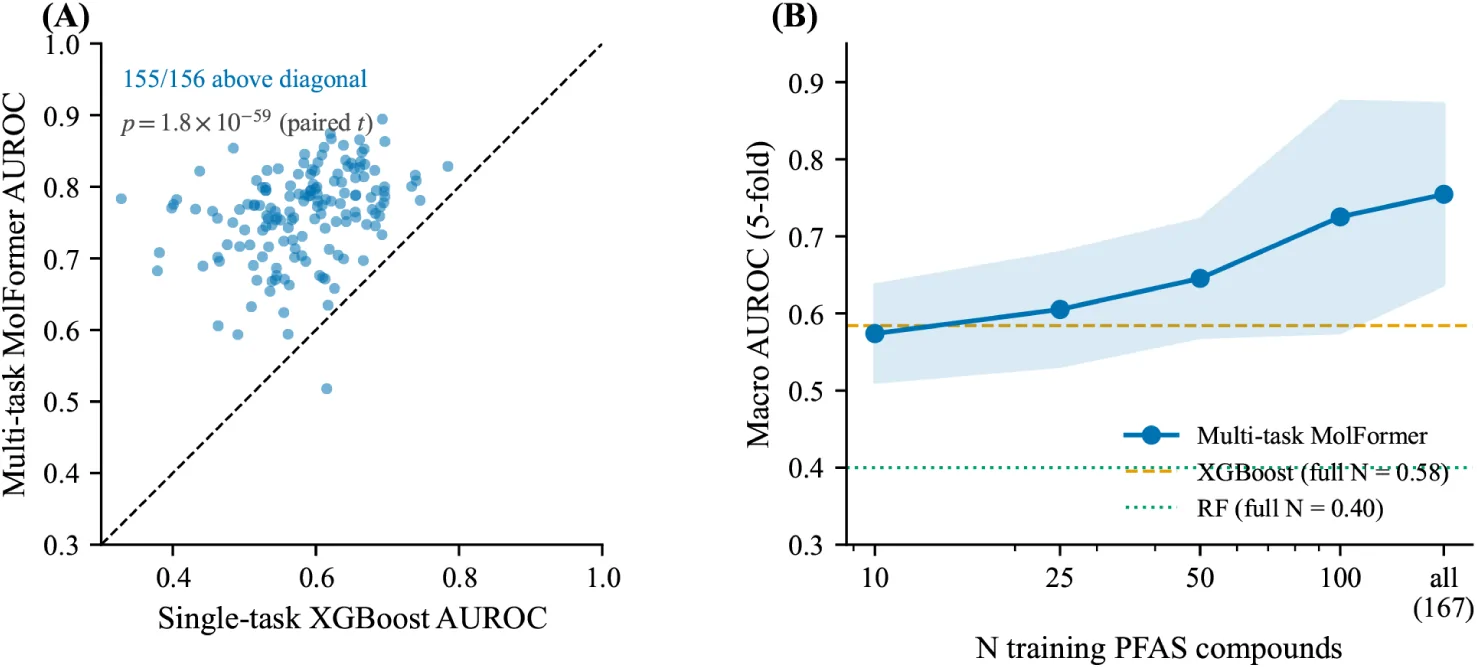
Off-the-shelf MolFormer-XL with 156 multitask heads vs single-task XGBoost on PFAS ToxCast bioactivity. (A) Per-end point AUROC scatter (multitask *y* vs XGBoost *x*); 155 of 156 points (99.4%) lie above the diagonal (mean Δ = +0.18). (B) Few-shot transfer curve (macro AUROC vs number of training PFAS; shaded band ± 1 std over 5 folds; dashed/dotted lines: full-*N* XGBoost and RF).

**Table 2:** Comparison of All Models Under 5-Fold Tanimoto-Butina Cluster-Out Cross-Validation on the PFAS ToxCast Benchmark^a^ ^,^ ^b^

Model	End points	Macro AUROC	Macro AUPRC	AUROC win rate
Morgan-FP + RF (single-task)	142	0.40 ± 0.09	0.46 ± 0.15	132/142
Morgan-FP + XGBoost (single-task)	156	0.58 ± 0.08	0.36 ± 0.10	155/156
Morgan-FP + MLP (multitask)	156	0.56 ± 0.08	0.34 ± 0.04	153/156
Graph neural network (multitask)	156	0.65 ± 0.10	0.43 ± 0.12	—
MolFormer multitask (aggr. DA)	156	0.75 ± 0.10	0.53 ± 0.17	—
MolFormer multitask (gentle DA)	156	0.74 ± 0.13	0.53 ± 0.21	—
**MolFormer multitask (off-the-shelf)**	156	**0.76 ± 0.10**	0.53 ± 0.17	—

a Macro AUROC and Macro AUPRC are Reported to Two Decimal Places as Mean ± Standard Deviation Across the 5 Folds; Bold Marks the Best Macro AUROC. AUROC Win Rate: Fraction of End Points on which the Off-The-Shelf Multi-Task MolFormer Exceeds the Listed baseline’s 5-Fold Mean AUROC (An End Point-Level Count).

b Off-the-shelf MolFormer is the headline model: + 0.18 macro AUROC (bootstrap 95% CI over end points [+0.17, + 0.19]) and +0.17 macro AUPRC over XGBoost, exceeding XGBoost on 155 of 156 end points. The end point-level paired comparison is descriptive because end points are not independent (Methods, “Reporting conventions”). The Morgan-FP + MLP (multitask) row is a control that isolates the learning paradigm from the representation: a multitask MLP on the *same* fingerprints (frozen-feature probe, identical to the Morgan cell of the factorial in the ablation below) reaches only 0.56 macro AUROC, below even the single-task XGBoost baseline (0.58) and far below the headline (0.76, won on 153/156 end points). Multitask learning on fingerprints alone therefore does not explain the foundation-model advantage. A multitask graph neural network (a GINE-style message-passing model trained from scratch on RDKit atom/bond features under the identical CV and multitask head) reaches 0.65 macro AUROC: above both fingerprint baselines but only at the level of the *frozen* MolFormer cell (0.64, Table 5) and well short of the fine-tuned headline (0.76). The advantage therefore comes from large-scale pretraining plus fine-tuning rather than from graph structure per se. RF is evaluable on 142 end points, because a class-balanced random forest cannot be fit on single-class training folds, so its macro AUPRC is computed over that subset only and is not directly comparable across rows.

This broad win is partly explained by single-task instability at small *N*. Binning the 142 RF-scorable end points by RF difficulty, 122 (86%) fall in a “degenerate” bin (RF mean AUROC < 0.50 or a majority of undefined folds), where the multitask model gains +0.39 mean ΔAUROC; the gain is +0.14 in the “hard” bin (RF 0.50–0.65, *n* = 15) and +0.06 in the “moderate” bin (*n* = 3), and only in the rare “easy” bin (RF ≥ 0.80, *n* = 2) does single-task RF marginally win (−0.14). The multitask system is thus far more stable on data-scarce PFAS end points. Because this headline comparison varies three factors together relative to the fingerprint baseline (representation, fine-tuning, and paradigm), it should be read as an end-to-end system comparison; the factorial ablation below disentangles the last two.

Figure 2B shows few-shot transfer. At *N* = 10 training PFAS the multitask model reaches macro AUROC 0.574 ± 0.063, essentially matching the full-*N* XGBoost baseline (0.584) with an order of magnitude fewer compounds. The area under the few-shot curve (AUFSC, log_10_ *N* grid {10, 25, 50, 100}) is 0.629 versus the constant XGBoost reference of 0.584.

#### Threshold-Dependent Metrics

Because the active class is rare, we repeat the headline-versus-baseline comparison at an explicit operating point. With the cut chosen on the training fold only, the multitask model reaches F1 0.432 ± 0.229, MCC 0.292 ± 0.258 and balanced accuracy 0.658 ± 0.135, against 0.202 ± 0.206, 0.110 ± 0.202 and 0.545 ± 0.084 for Morgan-FP plus XGBoost; at a fixed 0.5 cut the corresponding values are 0.256/0.202/0.583 versus 0.236/0.120/0.553. The ordering is the same as under AUROC and AUPRC, so the conclusion does not depend on the choice of metric family. The XGBoost arm is the one case where the training-fold-optimal cut transfers worse than a fixed 0.5 cut, an artifact of scale_pos_weight rebalancing driving the training-fold probabilities to a near-degenerate optimum; both rows are reported in Table S7 for transparency.

### The Fingerprint Baseline is Degenerate on Long-Chain PFAS, and Better Representations Close Part of the Gap

Binary Morgan fingerprints at a radius of 2 cannot resolve the perfluoroalkyl homologous series. On the 209 evaluation PFAS, **54 compounds (25.8%) share a bit-for-bit identical 2048-bit fingerprint with at least one other compound** in 21 collision groups of up to 8 members. Among the 32 carboxylic acid PFAS, 36 pairs have Tanimoto exactly 1.0 despite spanning perfluoroalkyl chain lengths from C5 to C13. The consequence is not merely aesthetic: within these collision groups there are 8,995 compound pairs labeled on a common end point, of which **3,316 (36.9%) carry opposite labels**, and every one of the 156 end points contains at least one such pair. Any model reading only this representation must therefore misclassify one member of each contradictory pair, which places a hard ceiling on the baseline, independent of the learner. Count-based Morgan fingerprints at either radius and the RDKit 2D descriptor block resolve all 209 compounds uniquely; MACCS keys are worse still (114 of 209 collide).

Table 3 therefore reruns the classical baselines across eight representations on the identical five folds. Three results matter. First, the sweep reproduces the submitted baseline (binary Morgan plus XGBoost, 0.589 here versus 0.584 in Table 2), confirming the two pipelines agree. Second, **the strongest classical representation is the RDKit 2D descriptor block at 0.688 ± 0.074**, so the margin properly attributable to the foundation model is +0.076, not the +0.18 measured against binary Morgan; we report both, and treat 0.688 as the honest comparator. Third, fixing the saturation matters most in the multitask setting: moving from binary to count Morgan lifts the multitask fingerprint MLP from 0.561 to 0.638, consistent with a shared representation being the binding constraint once end points are trained jointly. Conformer-based 3D descriptors do not help (0.603), so three-dimensional geometry is not the missing ingredient for this task.

**Table 3:** Molecular Representations Compared on the *Same* 5-Fold Tanimoto-Butina Split as Table 2 ^a^ ^,^ ^b^

	Representation	XGBoost (single-task)	MLP (multitask)
1D	Composition + chain length (12)	0.637 ± 0.052	0.610 ± 0.131
2D	Morgan bits *r* = 2 (submitted)	0.589 ± 0.029	0.561 ± 0.074
2D	Morgan bits *r* = 3	0.591 ± 0.059	0.575 ± 0.070
2D	Morgan counts *r* = 2	0.595 ± 0.096	0.638 ± 0.084
2D	MACCS keys (167)	0.544 ± 0.055	0.554 ± 0.084
2D	**RDKit 2D descriptors**	**0.688 ± 0.074**	0.658 ± 0.086
2D	RDKit 2D + Morgan counts	0.687 ± 0.073	0.683 ± 0.069
3D	Conformer shape + AUTOCORR3D	0.603 ± 0.045	0.580 ± 0.079
MolFormer-XL fine-tuned + multitask heads		**0.764 ± 0.100**	

a Macro AUROC, Mean ± SD Over Folds. “XGBoost” Is the B2 Protocol and “MLP Multi-Task” The B5 Protocol, Both Unchanged Except for the Input Features. Bold Marks the Best Classical Row.

b Compounds that no representation can distinguish set a ceiling on the learner: binary Morgan *r* = 2 maps 54 of 209 PFAS onto duplicate vectors, and 36.9% of the label pairs inside those groups disagree. Count Morgan, RDKit 2D descriptors, and the 3D block each resolve all 209 compounds.

### Label Missingness is Structured but Does Not Explain the Multi-Task Gain

Because ToxCast screening is organized around the EPA PFAS inventory rather than sampled at random, the label matrix could in principle manufacture a multitask benefit: compounds tested on many end points would be shared across many tasks, and joint training would exploit that coverage rather than any shared biology. We therefore characterize the missingness and test the confound directly.

The missingness is indeed strongly structured. Coverage is bimodal—146 of the 209 PFAS are screened on all 156 end points and 41 on fewer than ten—and it differs by subfamily (Kruskal–Wallis *H* = 22.3, *p* = 2.3 × 10^–3^, η^2^ = 0.098; Table S8), with PFECA and PFCA nearly fully screened (mean 155.9 and 143.8 end points) and the heterogeneous OTHER group least so (93.2). It is also aligned with chemical structure: the mean Jaccard similarity between two compounds’ label masks is 0.930 within a Butina cluster against 0.644 between clusters (difference +0.286, permutation *p* < 5 × 10^–4^, 2,000 permutations). Reviewer intuition on this point is correct, and it is a feature of ToxCast that PFAS modelers should expect.

It does not, however, propagate into the cross-validation or into the reported effect. The five cluster-out folds are balanced with respect to coverage (*H* = 7.41, *p* = 0.116, η^2^ = 0.026; within- versus between-fold mask similarity 0.652 vs 0.648), because the splitter balances cluster sizes across folds. More directly, the multitask advantage does not track cotesting: taking the fine-tuned single-task and multitask MolFormer arms, which differ only in paradigm, the mean gain is +0.071 on the half of end points whose compound sets overlap the other end points *least* and +0.092 on the most-overlapping half, with the multitask arm winning on 89% of end points in *both* halves. Finally, headline accuracy is not concentrated on the well-screened compounds: macro AUROC is 0.847 on the 63 partially screened PFAS and 0.780 on the 146 fully screened ones, the opposite of the direction the confound predicts. The partial stratum rests on 108 evaluable end point-folds against 776 for the full stratum, so we read this last comparison as directional rather than as a measured advantage.

### PFAS Domain Adaptation Contributes No Detectable Improvement

Neither Stage-1 domain-adaptation recipe improves over off-the-shelf fine-tuning (Figure 3, Table 4). The aggressive recipe (30 epochs, LR 5 × 10^–5^) gives Δ = – 0.012 macro AUROC (NS; paired *t p* = 0.67, Wilcoxon *p* = 0.81); the gentle recipe gives – 0.027 (NS; *p* = 0.43). Macro AUPRC agrees (both within +0.002 of off-the-shelf), and the off-the-shelf backbone wins on 93 of 156 end points (60%) vs aggressive DA. Two observations explain the null. First, MolFormer’s tokenizer already covers PFAS: it emits 208 out-of-vocabulary tokens in 1,089,670 tokens (0.019%) over the 11,028 unique PFASSTRUCT SMILES, and none at all on the 209 evaluation PFAS. Those SMILES draw on just 201 of the 2,362 vocabulary entries (8.5%), all standard ring, halogen, and stereo symbols abundant in the 1.1-billion-SMILES corpus. Second, the Stage-1 corpus (11,028 deduplicated PFAS) is 2 orders of magnitude smaller than the pretraining set, offering little new diversity. We frame this as a scoped negative result: it does not exclude benefits from substantially larger PFAS corpora, parameter-efficient adapters (e.g., LoRA), or other backbones (Discussion).

**Figure 3:**
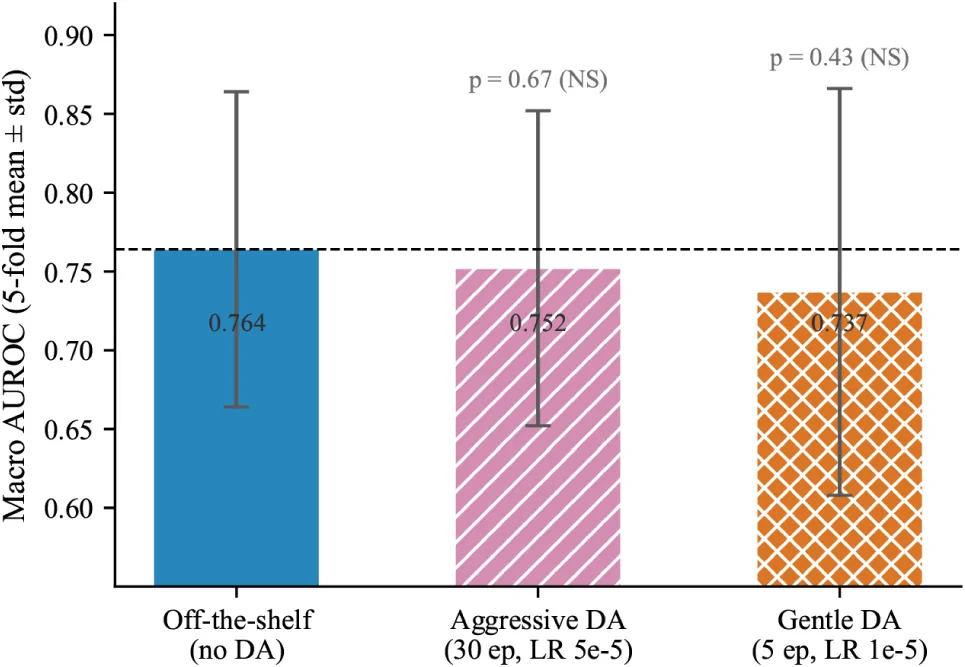
Stage-1 PFAS domain-adaptation ablation. Macro AUROC (mean ± std over 5 folds) for three backbone configurations on identical Butina splits. Neither the aggressive nor gentle recipe improves over off-the-shelf (NS, *p* > 0.40). Dashed line: off-the-shelf reference; bars hatched and value-labeled for grayscale readability.

**Table 4:** Stage-1 PFAS Domain-Adaptation Ablation^a^,^b^

Configuration	Macro AUROC	Δ*A*UROC	Macro AUPRC	Δ*A*UPRC
Off-the-shelf (no DA)	0.764 ± 0.100	—	0.530 ± 0.170	—
Aggressive DA (30 ep, LR 5 × 10^–5^)	0.752 ± 0.100	–0.012	0.531 ± 0.172	+0.001
Gentle DA (5 ep, LR 1 × 10^–5^)	0.737 ± 0.129	–0.027	0.532 ± 0.206	+0.002

a Both Recipes are Compared Against the Off-The-Shelf Backbone (No Domain Adaptation) on Identical 5-Fold Tanimoto-Butina Splits, on Both Macro AUROC and Macro AUPRC. Paired Statistics Use the 5 Folds as Observations (N = 5).

b On AUROC, neither recipe differs significantly from off-the-shelf (aggressive: paired *t p =* 0.67, Wilcoxon *p* = 0.81; gentle: *p* = 0.43, *p* = 0.81). On AUPRC the differences are likewise negligible (Δ ≤ + 0.002, far below the ± 0.17–0.21 fold-level spread), so the negative domain-adaptation result holds on both metrics. Per-fold AUROC (off-the-shelf/aggressive DA): fold 0, 0.825/0.794; fold 1, 0.775/0.777; fold 2, 0.716/0.745; fold 3, 0.572/0.571; fold 4, 0.896/0.873.

### Fine-Tuning and Multi-Task Learning are Synergistic

To separate the two factors internal to the foundation-model pipeline we run the matched 2 × 2 ablation defined in Methods: MolFormer-XL frozen or fine-tuned, crossed with single- or multitask heads, every cell sharing one head, optimizer, budget, and the 5 Butina folds (Table 5). The two factors are synergistic, not additive. On a frozen backbone the multitask paradigm gives no detectable benefit (−0.009 macro AUROC; paired *t p* = 0.33): joint training cannot improve a fixed representation. Fine-tuning a single-task model adds only +0.036. But the two together add +0.125 over the frozen single-task cell. The fine-tuning × paradigm interaction is +0.089 (95% CI [+0.05, + 0.13]; one-sample *t* over the 5 per-fold interaction contrasts, *p* = 0.012), which is as large as either main effect and positive on every fold. The cleanest single contrast, fine-tuned multitask vs fine-tuned single-task (identical recipe, differing only in paradigm), has the multitask model winning on every fold (+0.044 to +0.136; *p* = 0.008). The synergy is not specific to AUROC: the AUPRC paradigm effect is – 0.003 frozen versus +0.073 fine-tuned, an interaction of +0.076 that tracks the +0.089 AUROC value (Table 5). The mechanism is straightforward. Multitask learning helps by letting gradient signal from data-rich end points reshape a *shared* representation, which is impossible when that representation is frozen. The headline result therefore requires the combination, and neither component alone reproduces it. The pretrained representation also contributes: even the frozen single-task cell (0.647) already exceeds Morgan-FP + XGBoost (0.584).

**Table 5:** Matched 2 × 2 Ablation Over Backbone Fine-Tuning and Learning Paradigm for the MolFormer-XL Representation^a^,^b^

MolFormer-XL backbone	Single-task	Multitask
Frozen ([CLS] + head)	0.647/0.388	0.638/0.385
Fine-tuned	0.683/0.457	0.764/0.530

a All Four Cells Share an Identical Head Architecture, Optimizer, 50-Epoch Budget, Batch Size, and the 5-Fold Tanimoto-Butina Splits; the Only Difference is Whether the Backbone Weights are Updated. Each Cell Reports Macro AUROC/macro AUPRC, Mean Across the 5 Folds; Bold Marks the Headline Model.

b Measured effects (macro AUROC): backbone fine-tuning adds +0.036 under single-task training and +0.125 under multitask; the multitask paradigm adds – 0.009 (not significant, paired *t p =* 0.33) on a frozen backbone and +0.080 once it is fine-tuned. The fine-tuning × paradigm interaction is +0.089 (95% CI [+0.05, + 0.13]; one-sample *t* over the 5 per-fold contrasts, *p* = 0.012)—as large as either main effect: multitask learning yields no detectable benefit on a frozen backbone and requires a fine-tunable one. Macro AUPRC shows the same synergy: the paradigm effect is – 0.003 frozen vs +0.073 fine-tuned, an AUPRC interaction of +0.076 that mirrors the AUROC interaction. Even the frozen single-task cell (0.647 AUROC) exceeds the Morgan-FP + XGBoost baseline (0.584, Table 2), so the pretrained representation also contributes.

### The Synergy Reproduces Across Data Sets but is Backbone-Dependent

We reran the matched four-cell design along two axes (Table 7, whose footnote lists per-fold interactions): a *data set* axis (Tox21, SIDER, ClinTox under scaffold 3-fold CV) and a *backbone* axis (the PFAS task and all three MoleculeNet sets rerun with ChemBERTa-2, a RoBERTa encoder on a smaller, different corpus). All cells use the full headline budget; the MoleculeNet runs use 3 folds with no per-fold significance test and remain a qualitative check.

**Table 6:** Label-Free Encoder Diagnostics on the 209 Evaluation PFAS, Computed on Frozen Mean-Pooled Embeddings with No Bioactivity Labels^a^,^b^

Diagnostic	MolFormer-XL	ChemBERTa-2
Embedding dimension	768	384
Out-of-vocabulary rate on PFAS	0.000	0.000
Out-of-vocabulary rate on drug-like reference	0.000	0.000
Tokens per heavy atom (PFAS)	2.222	2.223
Distinct tokens used on PFAS/vocabulary size	31/2,362	19/591
Mean pairwise cosine (lower is less collapsed)	**0.875**	0.898
Effective rank of embedding covariance	13.4	10.5
Spearman (embedding vs Tanimoto distance)	0.608	**0.692**
Chain-length ridge probe *R* ^2^ (5-fold)	0.893	**0.920**
1-NN subfamily retrieval accuracy	0.612	**0.727**
PFAS macro AUROC, fine-tuned multitask	**0.74**	0.62

a A Drug-Like Reference Set of 1,000 Tox21 Compounds is Used for the Comparison Out-Of-Vocabulary Rate. Bold Marks the Better Value Where a Direction is Meaningful.

b The downstream ordering is not predicted by any static diagnostic: ChemBERTa-2 wins three of the three chemistry-recovery measures yet loses by 0.12 macro-AUROC after fine-tuning. Representation quality measured on a frozen encoder is therefore a poor guide to fine-tuned performance, which is itself a practical finding for backbone selection.

**Table 7:** The Matched 2 × 2 Ablation Across Two Independently Pretrained Backbones—molformer-XL (Linear Attention, 1.1-Billion-SMILES Corpus) and ChemBerta-2 (DeepChem/ChemBERTa-77M-MLM, RoBERTa, 77M-SMILES corpus)—on the PFAS Task and Three MoleculeNet Multi-Task Benchmarks^a^,^b^

		Frozen		Fine-tuned		
Backbone	Benchmark	ST	MT	ST	MT	Interaction
MolFormer-XL	PFAS (this work)	0.647	0.638	0.683	**0.764**	+0.089
MolFormer-XL	Tox21	0.741	0.747	0.688	0.769	+0.075
MolFormer-XL	SIDER	0.598	0.596	0.608	0.615	+0.009
MolFormer-XL	ClinTox	0.980	0.979	0.987	0.979	–0.007
ChemBERTa-2	PFAS	0.547	0.558	0.625	0.621	–0.015
ChemBERTa-2	Tox21	0.673	0.664	0.744	0.776	+0.041
ChemBERTa-2	SIDER	0.553	0.538	0.587	0.585	+0.014
ChemBERTa-2	ClinTox	0.854	0.847	0.989	0.988	+0.006

a All Cells Now Share the *Full* Headline Budget (50 Fine-Tuning Epochs, 5-Epoch Head Warm-Up); the MoleculeNet Runs Use Scaffold 3-Fold CV and the PFAS Runs 5-Fold Butina CV. The Interaction is the Fine-Tuning × Paradigm Contrast [paradigmft – paradigmfrozen]—the Gap in How Much Fine-Tuning Helps the Multi-Task Vs. Single-Task Head. The PFAS MolFormer-XL Row is the Headline Factorial (Table 5).

b Two readings emerge. (1) *Data set axis (MolFormer-XL):* the synergy reproduces on Tox21, positive on every fold ({+0.054, + 0.056, + 0.115}), with single-task fine-tuning now *degrading* below frozen features (0.688 < 0.741) while the multitask objective keeps fine-tuning a net gain; SIDER is weakly positive (+0.009, both heads ∼ 0.60, little fine-tuning headroom) and ClinTox is a null at the ∼ 0.98 ceiling. (2) *Backbone axis (PFAS task):* the synergy is *backbone-dependent*. ChemBERTa-2 reproduces it on Tox21 (+0.041; folds {+0.040, + 0.044, + 0.040}) but *not* on PFAS itself (−0.015; folds {+0.032, + 0.006, −0.055, −0.016, −0.042}), where fine-tuning lifts ChemBERTa-2 only 0.55 → 0.62, far short of MolFormer-XL, leaving no fine-tuning gain for multitask to compound. This is *not* attributable to poorer static encoding of PFAS structure (Table 6). Running the identical harness on PFAS with MolFormer-XL gives interaction +0.063 (vs the +0.089 headline from the dedicated 5-fold pipeline), so the MolFormer-vs-ChemBERTa contrast on PFAS is same-code. The synergy thus tracks whether backbone fine-tuning is itself effective for a given backbone × task, consistent with its mechanism, rather than holding universally.

Tox21 is the closest external benchmark to our task, which invites the question of whether it is independent of it at all. It is, on the axis that matters: none of our 156 ToxCast end points is a Tox21-sourced assay, and none of the 12 MoleculeNet Tox21 task names corresponds to any of them. The two data sets do share compounds—23 of the 209 PFAS (11.0%) appear among the 7,831 MoleculeNet Tox21 structures, or 0.3% of that benchmark—but this cannot create the interaction being measured, because every cell of the 2 × 2 sees exactly the same Tox21 data and the interaction is a within-benchmark contrast. Tox21 is used here precisely because it is the multitask, imbalanced, small-per-task setting most analogous to ours while being drawn from different assays.

On the *data set* axis the synergy is not a PFAS idiosyncrasy: for MolFormer-XL it reproduces on Tox21, the closest comparable benchmark, with the multitask paradigm inert on the frozen backbone (+0.006, like PFAS’s – 0.009) and helpful once fine-tuned (+0.081, like PFAS’s + 0.080), an interaction of +0.075 positive on all 3 folds. There, single-task fine-tuning even degrades below frozen features (0.688 < 0.741, overfitting 12 small heads) while the multitask objective keeps fine-tuning a net gain, the same mechanism seen on PFAS. SIDER is weakly positive (+0.009; both heads near 0.60 with little fine-tuning headroom) and ClinTox null (−0.007) at the ∼ 0.98 ceiling. The full budget cleans up two reduced-budget artifacts (an apparent SIDER sign-flip and a spuriously large ClinTox interaction), both traceable to under-trained frozen cells.

On the *backbone* axis the picture is sharper: the synergy is *backbone-dependent*. ChemBERTa-2 reproduces it on Tox21 (+0.041, positive on every fold), which reduces the concern that the effect is an artifact of MolFormer’s architecture or corpus. On the PFAS task itself, however, ChemBERTa-2 shows none (−0.015). The proximate cause is that fine-tuning simply buys ChemBERTa-2 less on PFAS (0.55 → 0.62, versus MolFormer’s 0.65 → 0.74), leaving little gain for the multitask objective to compound. Running the identical harness on PFAS with MolFormer-XL yields interaction +0.063 (vs the +0.089 dedicated-pipeline headline), confirming the contrast is same-code.

Why fine-tuning buys ChemBERTa-2 less is a separate question, and the obvious explanation—that ChemBERTa-2 simply encodes PFAS structure poorly—is *not* supported by the data (Tables 6 and 7). On every label-free diagnostic we can compute, ChemBERTa-2’s frozen representation is at least as PFAS-aware as MolFormer-XL’s: its embedding distances track Tanimoto distance more closely (Spearman 0.692 vs 0.608), a linear probe recovers perfluoroalkyl chain length from it slightly better (*R* ^2^ = 0.920 vs 0.893), and its nearest neighbors agree on subfamily more often (0.727 vs 0.612). Neither tokenizer has any PFAS vocabulary problem—both emit zero out-of-vocabulary tokens on the 209 evaluation PFAS at essentially identical token-per-heavy-atom rates—so lexical coverage does not distinguish them either. What these diagnostics do rule out is a static representation-quality explanation. The remaining candidate is capacity and fine-tunability: ChemBERTa-77 M is roughly an order of magnitude smaller than MolFormer-XL and its embedding space is lower-dimensional and slightly more collapsed (effective rank 10.5 of 384 dimensions vs 13.4 of 768). We did not test that hypothesis here—doing so would require a matched capacity sweep—and we flag it as the natural next experiment rather than as a finding. We therefore report the scope of the synergy empirically: it holds for MolFormer-XL on both PFAS and Tox21 and for ChemBERTa-2 on Tox21, and is absent for ChemBERTa-2 on PFAS, which is sufficient to rule out universality but not to explain the boundary.

### Embeddings Transfer to a Soil-Water Partitioning Regression Task

On the Fabregat-Palau 2025 *K*_d_ data set^8^ (Table 8), MolFormer [CLS] embeddings plus a Ridge head (refit per fold; backbone never exposed to log *K*_d_) achieve Spearman ρ = 0.79 ± 0.21 and *R* ^2^ = 0.59 ± 0.25 under 5-fold Butina CV, versus 0.59 ± 0.23 for Morgan-FP plus XGBoost (+0.20) and 0.52 ± 0.25 for RF; MAE is 0.46 vs 0.73 log units. This is first a cross-task transfer result (a property type never seen labeled). It is also a partial test on unseen chemistry: of the 42 PFAS, 23 overlap the 209 ToxCast PFAS and 19 do not. The leave-overlap-out test (Table 8, lower panel), which trains on the 23 in-distribution PFAS and tests on the 19 novel ones, holds MolFormer at ρ = 0.75 (95% CI [+0.37, + 0.94], *R* ^2^ = 0.58), essentially the full-CV value, whereas RF and XGBoost fall to ρ = 0.32/0.33 with CIs spanning zero and negative *R* ^2^. The representation transfers across both property *type* and unseen chemistry, though the small held-out *N* (wide CIs) keeps this directional rather than precise.

**Table 8:** Cross-Task Transfer to the Fabregat-Palau 2025 Soil-Water Partitioning (*K*_d_) Dataset: 42 PFAS Spanning 7 Subfamilies, 1,207 (PFAS, Soil) Log *K*_d_ Measurements Averaged per-PFAS^a^,^b^ ^8^

Model	Features	Spearman ρ	*R* ^2^	MAE (log *K*_d_)
*5-fold Tanimoto-Butina CV (all 42 PFAS)*				
Morgan-FP + RF	2048-bit, *r* = 2	0.52 ± 0.25	0.18 ± 0.22	0.68 ± 0.14
Morgan-FP + XGBoost	2048-bit, *r* = 2	0.59 ± 0.23	0.09 ± 0.30	0.73 ± 0.13
MolFormer + RidgeCV	**768-d [CLS]**	**0.79 ± 0.21**	**0.59 ± 0.25**	**0.46 ± 0.17**
*Leave-overlap-out (tes*t = 19 *unseen-chemistry PFAS)*				
Morgan-FP + RF	2048-bit, *r* = 2	+0.32	–0.03	0.83
Morgan-FP + XGBoost	2048-bit, *r* = 2	+0.33	–0.91	1.15
MolFormer + RidgeCV	**768-d [CLS]**	**+0.75**	**+0.58**	**0.50**

a Evaluation Under 5-Fold Tanimoto-Butina Cluster-Out CV. The MolFormer Backbone was Never Trained on Log Kd; Only a 768 → 1 Ridge Regression Head was Fit per Fold. Values: Mean ± Standard Deviation Across 5 Folds (Two Decimal Places); Bold Marks the Best Model. The Lower Panel is the Leave-Overlap-Out Test: A Single Split Training on the 23 PFAS That Share a Structure with the ToxCast Set and Testing on the 19 Whose Chemistry is Unseen There.

b Of the 42 *K*_d_ PFASs, 23 share a DTXSID or canonical SMILES with the 209 ToxCast PFASs and 19 do not. In the leave-overlap-out test, the model is trained on the 23 in-distribution PFAS and evaluated on the 19 structurally novel ones; bootstrap 95% CIs over the 19 test points are [−0.23, + 0.71] (RF), [−0.22, + 0.72] (XGBoost), and [+0.37, + 0.94] (MolFormer). Only MolFormer retains a clearly positive correlation on unseen chemistry; both fingerprint baselines collapse to a CI spanning zero with a negative *R* ^2^. The small held-out *N* is reflected in the wide CIs; therefore, this is a directional check rather than a precise effect size.

### Mondrian Conformal Calibration Improves Positive-Class Coverage

Table 9 compares three calibration protocols (Methods; detail in Section S6). Vanilla split conformal at target marginal 0.90 achieves 0.870 marginal coverage but only **0.363 on the active class**, which is wrong with confidence on 63% of true positives, a serious failure for screening where missing an active compound is costly, masked by an apparently high 84.5% singleton rate. Mondrian-by-class calibration corrects this: under the recycled-fold protocol (where the larger calibration set permits data-driven quantiles) it raises active-class coverage to **0.778** while holding marginal coverage at 0.930, at the cost of more abstention (56% two-element sets). The exchangeability-respecting true-holdout protocol (1,000 evaluations over 5 folds × 200 splits) gives marginal 0.925 ± 0.054 and positive-class 0.914 ± 0.077, but at *n*_cal_ ≈ 20 a conservative fallback fires for 74% of end points, so these figures are fallback-inflated. Pooling all 5 models’ out-of-fold predictions (median 83 calibration compounds, fallback 74% → 1%) still holds marginal at 0.923 and positive-class at 0.934, confirming the positive-class gain is genuine rather than a fallback artifact, under approximate cross-fold exchangeability. We treat the recycled-fold result as substantive, the other two as consistency checks, and present conformal calibration as exploratory rather than coverage-guaranteed (Figure 4).

**Figure 4:**
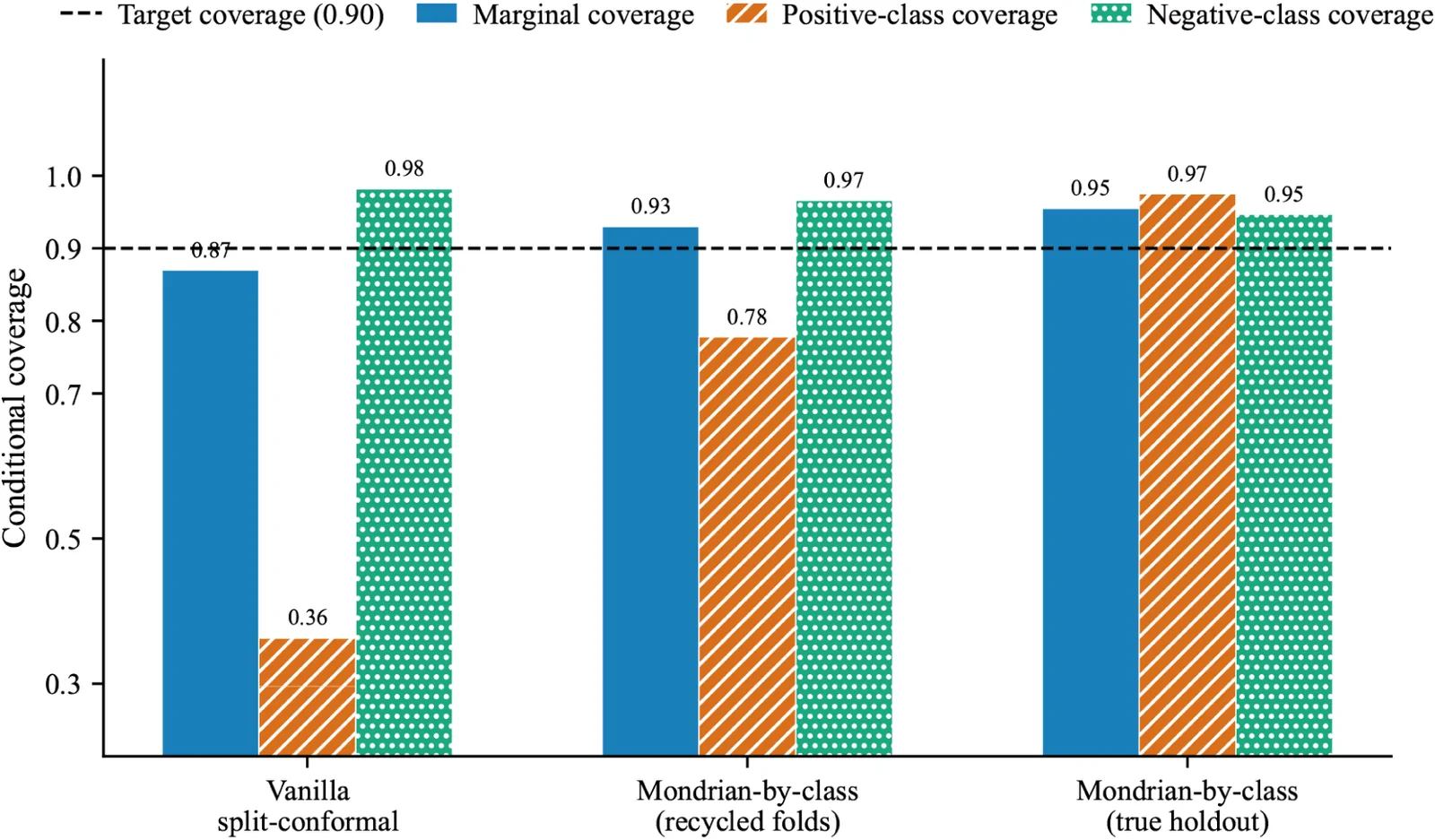
Conformal coverage at target marginal 0.90 (dashed line). Vanilla split conformal (left) meets the marginal target but covers only 36% of true positives. Mondrian-by-class (center, recycled-fold) raises positive-class coverage to 0.78; the right-hand true-holdout bars reach higher coverage but are fallback-dominated at small calibration size. Bars distinguished by color and hatch: marginal (blue, solid), positive (vermilion, diagonal), negative (green, dotted).

**Table 9:** Conformal Prediction Coverage Metrics at Target Marginal Coverage 1 – *α* = 0.90^a^,^b^

Method	Marginal	Pos.-class	Neg.-class	Avg set size	Singleton (%)
Vanilla split-conformal	0.870	0.363	0.982	1.155	84.5
**Mondrian-by-class (recycled)**	**0.930**	**0.778**	**0.966**	**1.561**	**43.9**
Mondrian-by-class (true holdout)	0.925	0.914	0.926	1.620	37.9
Mondrian-by-class (pooled holdout)	0.923	0.934	0.919	1.700	30.0

a “Recycled Folds” Uses Folds 0–2 as Calibration and Folds 3–4 as Test (Calibration and Test Compounds Not Strictly Exchangeable). “True Holdout” Is the Exchangeability-Respecting Protocol, Here Run on All 5 Folds with 200 Random 50/50 Calibration/Test Splits Each (1,000 Evaluations). “Pooled Holdout” Calibrates on the Pooled Out-Of-Fold Predictions of All 5 Models (209 Compound-Rows; 200 Random 50/50 Splits), Giving a ∼ 4× Larger Per-End Point Calibration Set. Avg. Set Size: 1 = Singleton (Confident Prediction); 2 = Abstention ({active, Inactive}). All Metrics are Macro Averages Over 156 End Points.

b Target: marginal coverage = 0.90. Vanilla met the marginal target but covered only 36% of true positives. The recycled-fold Mondrian row (bold) is the substantive result: its larger calibration set permits data-driven class quantiles. The true-holdout row is the mean over the 1,000 repeated splits (marginal SD 0.054, positive-class SD 0.077); at *n*_cal_ ≈ 20 the conservative class-quantile fallback fires for 74% of end points, so its coverage is fallback-influenced. The pooled-holdout row escapes that regime: pooling out-of-fold predictions raises the median per-end point calibration count to ∼ 83 and drops the fallback rate to 1%, yet marginal (0.923) and positive-class (0.934) coverage hold at or above target—so the calibration conclusion is not an artifact of the conservative fallback. Pooling mixes predictions from 5 fold-models, so its exchangeability is approximate (the standard cross-conformal compromise).

### The Result Does Not Depend on One Partition

With 209 compounds, a single 5-fold partition is a legitimate concern. Repeating the entire protocol under four additional split seeds—each regenerating the Butina partition and retraining the headline model from scratch—gives macro AUROC 0.764, 0.738, 0.771, 0.734 and 0.748, i.e., 0.751 ± 0.014 across partitions (range 0.734–0.771). The across-partition spread is thus roughly seven times smaller than the within-partition fold-to-fold spread (±0.10 for the reported seed; across all five partitions individual folds range from 0.58 to 0.88). The headline number is therefore a property of the data rather than of a fortunate split, while individual per-fold values should not be overinterpreted. This is consistent with the cutoff sweep (Table S5), where varying the Butina threshold over {0.5, 0.6, 0.7, 0.8} leaves the partition essentially unchanged.

### Chemical Space and Applicability Domain

The cross-validation clusters are internal to PFAS chemistry, and it is worth confirming that they are not merely separating PFAS from non-PFAS. Projecting Morgan-count space with t-SNE^22^ over the 209 evaluation PFAS, 1,500 PFAS sampled from the wider PFASSTRUCT library, and 1,500 non-PFAS Tox21 compounds (Figure 5) shows the two chemistries occupying essentially disjoint regions: a compound’s nearest neighbor shares its PFAS/non-PFAS status 95.9% of the time. The cluster-out folds and the subfamily labels, by contrast, interleave *within* the PFAS region (Figure 5b,c), which is the intended behavior—the folds separate structurally similar PFAS from one another rather than exploiting a PFAS-versus-drug-like boundary.

**Figure 5:**
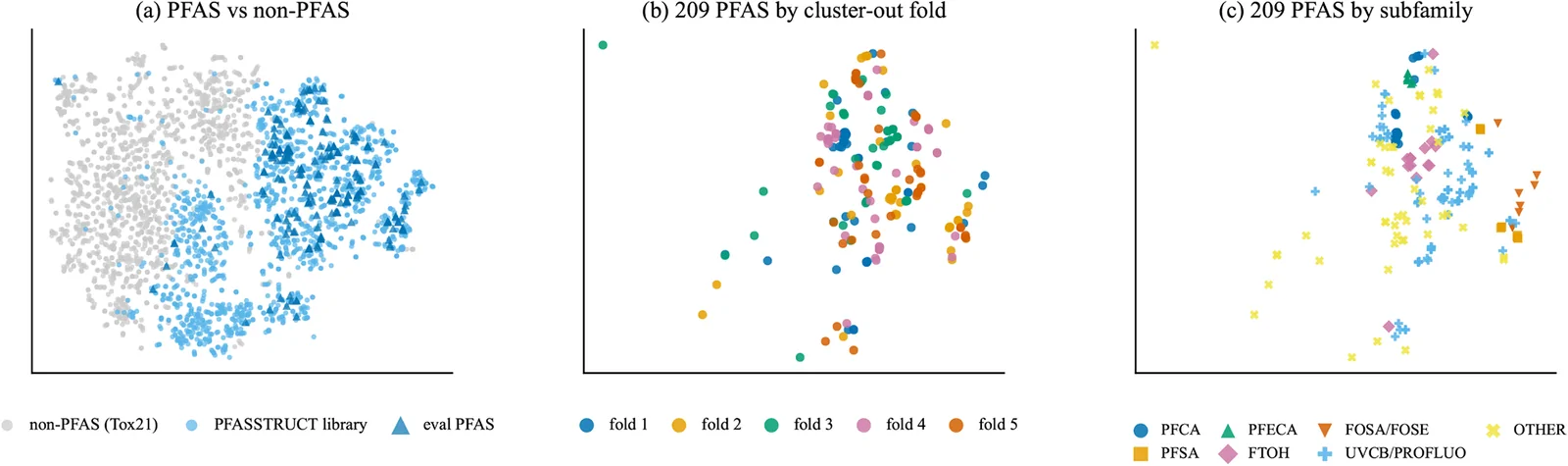
Chemical space of the benchmark (t-SNE of 2048-bit Morgan space, Jaccard metric). (a) The 209 evaluation PFAS and 1,500 sampled PFASSTRUCT library PFAS against 1,500 non-PFAS Tox21 compounds; nearest-neighbor PFAS/non-PFAS purity is 0.959. (b) The 209 evaluation PFAS colored by Tanimoto-Butina cluster-out fold; folds interleave within the PFAS region rather than tracking the PFAS/non-PFAS boundary. (c) The same compounds colored by subfamily.

#### Applicability Domain

Defining the distance to the model’s domain as the mean Tanimoto similarity to the five nearest training-fold compounds, accuracy is remarkably flat across quartiles of that distance: macro AUROC 0.737, 0.724, 0.730, and 0.759 from the least to the most similar quartile (Figure 6a). Cluster-out CV has already pushed every held-out compound away from its training neighbors—the median held-out compound sits at a mean-top-5 Tanimoto of about 0.45—so within the range the benchmark spans there is no detectable decay. This is a narrower statement than a conventional applicability domain, and it should not be read as license to extrapolate.

**Figure 6:**
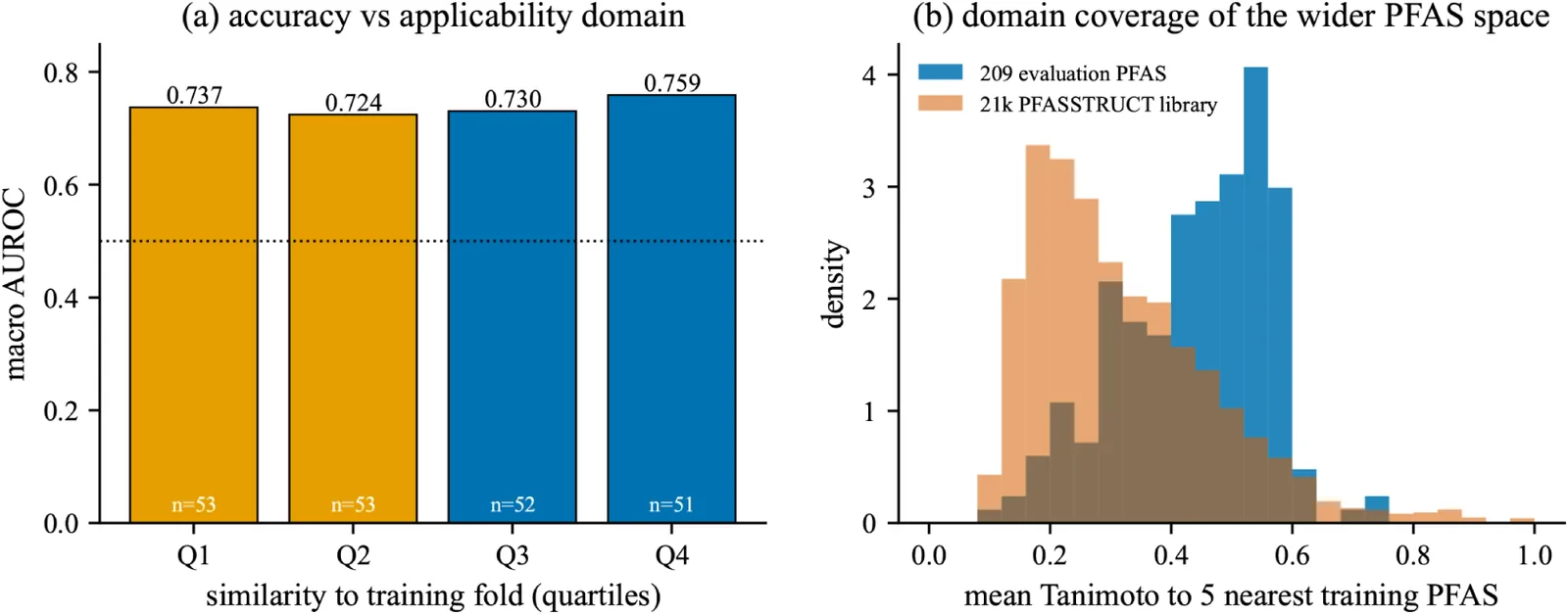
Applicability domain. (a) Macro AUROC of the headline model by quartile of similarity to the training fold (mean Tanimoto to the 5 nearest training compounds); accuracy does not decay across the range the benchmark spans. (b) Distribution of that similarity for the 209 evaluation PFAS and for the 20,793 untested PFASSTRUCT compounds; most of the library lies further from the training set than any evaluated compound.

The practical limit appears when the same measure is applied outward. Of the 20,793 PFASSTRUCT compounds outside the evaluation set, only 46.9% lie within a mean-top-5 Tanimoto of 0.3 of the training compounds, 26.2% within 0.4, and 12.2% within 0.5 (Figure 6b). A model trained on 209 compounds therefore covers a minority of the known PFAS universe at any defensible similarity threshold. We recommend reporting this distance alongside any prediction and treating compounds beyond a mean-top-5 Tanimoto of 0.3–0.4 as outside the evaluated domain.

### Model Performance is Strongly Subfamily-Dependent

Per-subfamily macro AUROC from the 5-fold Butina predictions (Figure 7) ranges widely. PFECA (GenX/HFPO–DA family, *n* = 7) is the most predictable (0.906), likely because these short ether-carboxylic acids are well separated from other clusters. FOSA/FOSE sulfonamides (*n* = 8) fall to 0.427, below random, reflecting both the rarity of sulfonamide labels and their structural dissimilarity from the predominantly perfluoroalkyl acid training compounds. The Mondrian model correctly abstains from most of them, which shows that calibration tracks actual difficulty.

**Figure 7:**
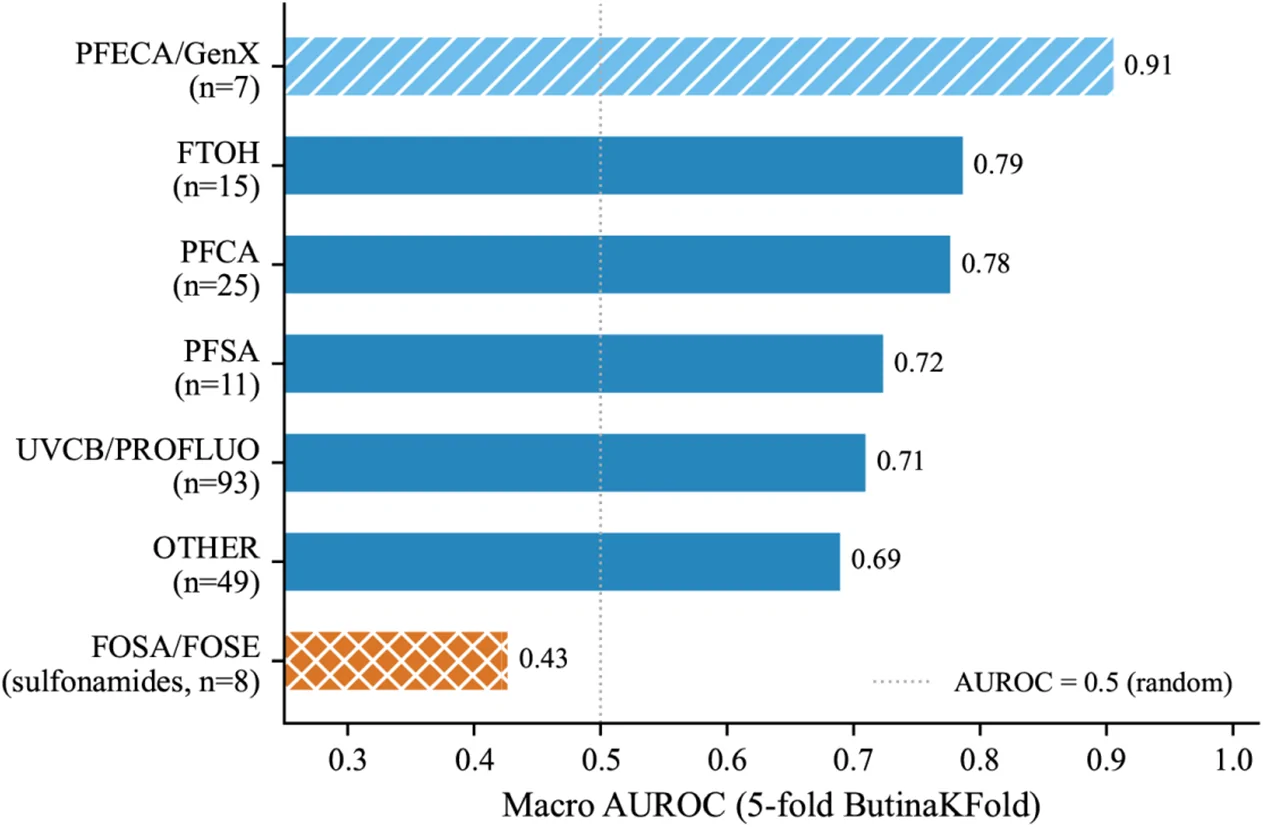
Per-subfamily macro AUROC on 5-fold Butina cluster-out predictions. PFECA (sky blue, diagonal hatch) is most predictable; FOSA/FOSE (vermilion, cross hatch) are systematically misclassified (below 0.5). Bars annotated with values for grayscale; dotted line AUROC = 0.5. These are in-distribution per-subfamily accuracies under generic Butina folds; Table 10 reports the held-out-subfamily extrapolation.

These per-subfamily numbers allow members of each subfamily into the training folds, so they measure in-distribution accuracy. For true extrapolation we run leave-one-subfamily out CV: each subfamily is held out *entirely* and the matched multitask model is fine-tuned on the rest (Table 10; full values in Table S3). Against the matched in-distribution mean (0.717), extrapolation to a wholly unseen subfamily costs – 0.018 macro AUROC unweighted and – 0.044 compound-weighted, the larger weighted gap reflecting the big, heterogeneous UVCB_PROFLUO class (0.71 → 0.59 over 93 PFAS). Most subfamilies extrapolate well (0.73–0.82), the well-separated PFECA ether-acids drop as expected (0.91 → 0.79), and the two failures are interpretable (UVCB_PROFLUO and FOSA/FOSE at 0.412, already the weakest in-distribution). That the model still reaches ∼ 0.70 on unseen chemistries indicates reliance on broad structural features rather than subfamily specific memorization. Because the per-subfamily OOD estimates are single noisy evaluations over *n* = 7–93 compounds, we report the mean drop rather than any single Δ.

**Table 10:** True Out-Of-Distribution Extrapolation: Leave-One-Subfamily-Out CV^a^,^b^

Held-out subfamily	*n*	In-distribution	Subfamily out (OOD)	Δ
PFECA (GenX/HFPO–DA)	7	0.906	0.791	–0.115
FTOH	15	0.787	0.816	+0.029
PFCA	25	0.777	0.794	+0.017
PFSA	11	0.724	0.764	+0.040
OTHER	49	0.690	0.728	+0.038
UVCB_PROFLUO	93	0.710	0.588	–0.122
FOSA/FOSE	8	0.427	0.412	–0.015
Mean (unweighted)	–	0.717	0.699	–0.018
Mean (compound-weighted)	–	0.715	**0.672**	–0.044

a Each PFAS Subfamily is Held Out *entirely*, the Matched Multi-Task MolFormer-XL is Fine-Tuned (Headline Recipe) on All Other Subfamilies, and Evaluated on the Held-Out Subfamily. “In-Distribution” Is the Per-Subfamily Macro AUROC Under Generic 5-Fold Butina CV (Figure 7), Where Each Subfamily Has Members in the Training Folds. Δ is the Out-Of-Distribution Change. PFESA (N = 1) is Omitted as Unevaluable.

b Compared against the *matched* per-subfamily in-distribution mean (0.717 unweighted), genuine chemistry extrapolation costs – 0.018 unweighted and – 0.044 when weighted by compound count; the larger weighted drop reflects the big, heterogeneous UVCB_PROFLUO class (93 PFAS, 0.71 → 0.59). Most subfamilies extrapolate well (0.73–0.82); the two clear failures are interpretable—UVCB_PROFLUO and the perfluoroalkyl sulfonamides (FOSA/FOSE, 0.412, already the weakest class in-distribution)—and the well-separated PFECA ether-acids drop as expected (0.91→0.79). Each subfamily is a single held-out evaluation over *n* = 7–93 compounds with no per-subfamily uncertainty estimate, so individual values (especially for the small classes) are noisy; we read the mean drop, not any single Δ, as the OOD summary.

## Discussion

### Why Multitask Transfer Works for PFAS

The broad win over single-task baselines stems from two mechanisms. The shared backbone propagates information across correlated end points: the 156 ToxCast assays are concentrated in a few phenotypic readout families—71 of 156 are cytokine end points and 31 are cell-adhesion-molecule end points, largely from the same primary-cell panels—so they measure overlapping inflammatory and cell-state responses, and gradient signal from data-rich end points regularizes data-scarce ones. That concentration is a double-edged property: it plausibly explains why joint training transfers so readily here, and it also limits how far the result should be extrapolated to a panel of mechanistically independent assays. Separately, MolFormer’s pretrained representation captures global structure (chain length, fluorination, headgroup chemistry) that 2048-bit Morgan fingerprints represent only locally. The difficulty-bin analysis confirms the advantage is largest (+0.39) precisely in the degenerate bin where single-task models are unreliable. The factorial ablation makes this precise: on a frozen backbone multitask adds nothing (−0.009), because shared gradient cannot reshape a fixed representation; it helps (+0.08) only once the backbone is fine-tunable. Multitask transfer for PFAS is thus not free-standing but synergistic with a fine-tunable backbone. Rerunning the factorial along two axes sharpens its scope: on the *data set* axis it reproduces for MolFormer-XL on Tox21 (+0.075, all 3 folds), is weakly positive on SIDER, and null at the ClinTox ceiling. On the *backbone* axis it is backbone-dependent: ChemBERTa-2 reproduces the positive Tox21 interaction (+0.041) but shows none on PFAS (−0.015), because it is a weak PFAS encoder (0.55 → 0.62) whose small fine-tuning gain leaves little for multitask to compound. The interaction therefore appears wherever backbone fine-tuning is itself effective and is absent where it is not. This is an empirical scope, not a universal property of foundation models.

### Why Domain Adaptation Does Not Help

The negative Stage-1 result follows from the tokenizer analysis: the 1.1-billion-SMILES corpus yields near-zero out-of-vocabulary tokens on PFAS (0.019% over the 11,028-SMILES Stage-1 corpus, zero over the 209 evaluation compounds), which use only 201 of 2,362 vocabulary tokens (8.5%), all standard chemical symbols whose co-occurrence statistics are already learned from the full corpus; the 11,028-molecule Stage-1 corpus adds no diversity beyond ZINC and PubChem. A low out-of-vocabulary rate is evidence of *lexical* coverage only: it shows that PFAS SMILES tokenize without loss, not that the representation is optimal for bioactivity. The result is therefore best read as “further narrow-corpus MLM pretraining does not help here,” not as “the representation cannot be improved.” Practically: when starting from a broadly pretrained backbone, domain-specific SMILES pretraining on a narrow class (at the ∼ 11k-molecule scale tested) is unlikely to help unless the target chemistry is genuinely out-of-vocabulary; we cannot exclude that a much larger or more diverse PFAS corpus would behave differently.

### What is New Here, and What is Not

Neither the backbone nor multitask learning is novel: MolFormer-XL is used off-the-shelf and unmodified, and joint training across assays dates to massively multitask networks^5^ and multitask Tox21 models.^6^ What this study contributes is a controlled answer, for a chemical class where none existed, to which ingredient actually pays. Three of those answers were not predictable from the literature. First, the field’s default fingerprint baseline is not merely weak for PFAS but partly degenerate: a quarter of our compounds are indistinguishable under binary Morgan *r* = 2, and 36.9% of the label pairs inside those collision groups disagree, so a substantial part of the headline margin previously attributed to the foundation model was in fact a representation artifact—replacing the representation alone recovers more than half of it (Table eftab:representations). Second, domain-adaptive pretraining, the obvious move for a niche chemistry and a reliable win in NLP,^24^ is a no-op here under two very different recipes. Third, the fine-tuning*imes*multitask interaction that does carry the result is backbone-dependent in a way that frozen-embedding diagnostics do not anticipate. Reporting all three under one leakage-aware protocol, with the comparators fixed, is the contribution; the individual components are standard by design, because the point is to establish what a practitioner should expect from them.

### Methodological Generalizability

The ButinaKFold autofallback should apply beyond PFAS to other linear-chain-dominated classes (short-chain organic acids, aliphatic pesticides, amino-acid analogues) exhibiting the same scaffold collapse, though we validate it only on PFAS; we offer the 30-scaffold threshold as a practical untuned trigger. The Mondrian-by-class result likewise generalizes: any imbalanced binary bioactivity data set (≤20% active) is liable to severe positive-class under-coverage under vanilla conformal prediction.

### Limitations

*(i)* All 156 evaluation end points are ToxCast in vitro assays; in vivo toxicity, environmental fate, and clinical outcomes are not represented, and the *K*_d_ transfer, while encouraging, does not substitute for in vivo validation. *(ii)* The fine-tuning × multitask synergy is backbone-dependent and the negative domain-adaptation result is MolFormer-only: on a second backbone (ChemBERTa-2) the synergy reproduces on Tox21 but is absent on PFAS, so it is established firmly only for MolFormer-XL and should not be assumed for arbitrary backbones, and whether the DA null holds for ChemBERTa-2 or graph backbones needs separate experiments. *(iii)* LoRA-based Stage-1 adaptation was not tested and might adapt with less catastrophic forgetting. *(iv)* OOD performance varies sharply by subfamily: under leave-one-subfamily out CV the mean held-out AUROC is 0.699, but UVCB_PROFLUO (0.588) and FOSA/FOSE (0.412) extrapolate poorly and several subfamilies have few compounds (*n* ≤ 11), so OOD estimates on these chemistries are noisy and should be treated cautiously. *(v)* The 30-scaffold threshold and 0.6 Butina cutoff use community-standard values and were not sensitivity-tested for downstream performance; results may shift modestly under other settings. *(vi)* The benchmark contains 209 compounds, a small and nonrepresentative sample of the more than 21,000 known PFAS. It is the complete set for which ToxCast supplies usable binary hitcalls rather than a selection we made, but the limitation is real and quantifiable: only 26.2% of the 20,793 untested PFASSTRUCT compounds lie within a mean-top-5 Tanimoto of 0.4 of our training compounds (applicability domain). Conclusions should therefore not be extended to PFAS chemistries far outside this domain, and per-compound predictions on such chemistries should be flagged or withheld. Sample size also bounds what the baselines can show: 209 compounds across five folds is a regime in which tree ensembles and a from-scratch graph network are all data-starved. That is precisely the regime that motivates a pretrained backbone, but it means the baseline ordering reported here is an ordering *at this scale* and could change with substantially more labeled PFAS. *(vii)* Label missingness in ToxCast is structured by assay-panel design (missingness analysis); we show it does not explain the multitask gain in these data, but any extension of this benchmark should recheck that.

## Implications

For PFAS hazard screening, these results carry three practical implications. First, a broadly pretrained chemical foundation model fine-tuned with multitask heads is a strong, data-efficient default: off-the-shelf MolFormer-XL beats single-task fingerprint baselines on 155 of 156 ToxCast end points (+0.18 macro AUROC) and remains useful at *N* = 10 training compounds, the regime relevant to rare PFAS subfamilies. Narrow-corpus PFAS domain pretraining (two MLM recipes on PFASSTRUCT V6) adds nothing detectable, so practitioners should not expect gains from small in-domain pretraining when the backbone’s tokenizer already covers the chemistry (here, 0.019% unknown tokens). Second, Murcko scaffold cross-validation is degenerate for predominantly linear chemistries, since 187 of our 209 evaluation PFAS have an empty scaffold, so the Tanimoto-Butina autofallback we specify is a simple, reusable correction for any linear-chain-dominated data set and not only for PFAS. Third, Mondrian-by-class conformal calibration restores the positive-class coverage that vanilla conformal prediction loses on imbalanced toxicity end points (0.36 → 0.78), and is worth adopting wherever PFAS predictions feed decision support. These findings are backbone- and corpus-bounded: the fine-tuning × multitask synergy and the negative domain-adaptation result are established most firmly for MolFormer-XL. Larger and more diverse PFAS pretraining corpora, parameter-efficient (e.g., LoRA) adapters, graph foundation-model backbones, and extension to in vivo end points and environmental fate parameters beyond soil-water *K*_d_ remain the open directions for turning this benchmark into a deployable screening tool.

## Supplementary Material



## Data Availability

**Data and Software Availability** ToxCast invitroDB v4.3 and the PFASSTRUCT V6 list are publicly available from the U.S. EPA CompTox dashboard; the soil-water *K*_d_ data are in the Supporting Information of ref 8. The MolFormer-XL checkpoint (ibm/MoLFormer-XL-both-10pct) is publicly available from the Hugging Face Hub. Code for the Murcko-to-Butina cross-validation splitter, multitask training, the single-task and tree baselines, the factorial ablation, and the conformal calibration, together with the representation sweep, missingness, applicability-domain, threshold-metric, repeated-cross-validation and encoder-diagnostic analyses added in this revision, will be made available at https://github.com/zechariahyue/pfas-foundation-model on acceptance.

## Notes

per-end point AUROC/AUPRC for all 156 ToxCast end points (Table S1); the leave-overlap-out chemistry split for the *K*_d_ transfer test, with DTXSID and SMILES (Table S2); per-subfamily out-of-distribution results (Table S3); subfamily assignments with SMILES for all 209 PFAS (Table S4); the Butina-cutoff split-stability check (Table S5); conformal-calibration detail (Table S6); threshold-dependent metrics (F1, MCC, balanced accuracy; Table S7); the structured-missingness analysis (Table S8); the full eight-way representation sweep with AUPRC (Table S9); applicability-domain bins and library coverage (Table S10); repeated cross-validation over five independent partitions (Table S11); and a catalog of all 156 end points with assay metadata and PFAS counts, with a roll-up by intended target family (Tables S12 and S13) (PDF)
