# Supplementary material for: A Chemical Foundation Model Benchmark for PFAS Bioactivity Prediction: Multi-Task Fine-Tuning Beats Fingerprint Baselines, PFAS Domain Pretraining Does Not Help
Source: J Chem Inf Model. 2026 Sep 24;66(19):12518–33. doi: 10.1021/acs.jcim.6c02202 (PMC13663850; doi:10.1021/acs.jcim.6c02202)
Supplement: Supplementary file 1 [file ci6c02202_si_001.pdf]

Supporting Information: A Chemical Foundation Model Benchmark for PFAS Bioactivity Prediction: Multi-Task Fine-Tuning Beats Fingerprint Baselines, PFAS Domain Pretraining Does Not Help

Yue Zhu*,†,¶ and Qingyang Liu‡,¶

†*College of Computing, Georgia Institute of Technology, 225 North Avenue NW, Atlanta, GA 30332, USA*

‡*Khoury College of Computer Sciences, Northeastern University, 360 Huntington Avenue, Boston, MA 02115, USA*

¶*These authors contributed equally to this work.*

E-mail: yzhu766@gatech.edu

This document contains supporting tables for the main text. Table and section labels are prefixed with "S". All values are reproduced from the released result artifacts; no additional model training was performed for this Supporting Information except the deterministic Butina-cutoff split reproduction in Section S5.

S1. Per-endpoint performance (156 ToxCast endpoints)

Per-endpoint AUROC and AUPRC for the headline off-the-shelf MolFormer multi-task model, averaged over the 5 Tanimoto-Butina folds (Table S1).

Table S1: Per-endpoint performance of the off-the-shelf MolFormer multi-task model (headline) across the 156 ToxCast endpoints: mean AUROC and AUPRC over the 5 Tanimoto-Butina folds, with the number of folds in which the endpoint was evaluable (a fold is dropped when the test set has fewer than 5 labelled compounds or is single-class). Sorted by mean AUROC.

Endpoint	mean AUROC	mean AUPRC	folds
aeid_2827	0.895	0.588	5
aeid_2881	0.874	0.666	5
aeid_2895	0.867	0.635	5
aeid_2905	0.866	0.565	5
aeid_318	0.863	0.590	5
aeid_2891	0.858	0.602	4
aeid_292	0.855	0.577	5
aeid_2877	0.854	0.511	5
aeid_2929	0.853	0.566	5
aeid_260	0.848	0.574	5
aeid_2873	0.846	0.500	5
aeid_2833	0.845	0.614	5
aeid_2831	0.837	0.649	5
aeid_190	0.835	0.679	5
aeid_174	0.834	0.542	5
aeid_178	0.833	0.586	5
aeid_2879	0.833	0.528	5
aeid_2855	0.831	0.544	5
aeid_226	0.829	0.474	5
aeid_160	0.829	0.679	5
aeid_270	0.827	0.512	5
aeid_186	0.826	0.605	5
aeid_298	0.825	0.590	5

Endpoint	mean AUROC	mean AUPRC	folds
aeid_2829	0.824	0.651	5
aeid_264	0.823	0.591	5
aeid_296	0.823	0.628	5
aeid_250	0.822	0.603	5
aeid_2865	0.822	0.380	5
aeid_2851	0.818	0.605	5
aeid_280	0.818	0.591	5
aeid_2913	0.817	0.623	5
aeid_204	0.816	0.570	5
aeid_2917	0.814	0.608	5
aeid_2843	0.813	0.544	5
aeid_2919	0.809	0.622	5
aeid_274	0.808	0.599	5
aeid_2897	0.808	0.518	5
aeid_2845	0.806	0.559	5
aeid_2907	0.803	0.670	5
aeid_310	0.801	0.565	5
aeid_192	0.800	0.494	5
aeid_276	0.800	0.454	5
aeid_2853	0.800	0.541	5
aeid_182	0.799	0.575	5
aeid_3211	0.799	0.425	5
aeid_288	0.797	0.562	5
aeid_312	0.795	0.417	5
aeid_2819	0.795	0.557	5
aeid_196	0.795	0.592	5
aeid_2821	0.795	0.492	5
aeid_2935	0.794	0.636	5
aeid_2875	0.794	0.592	5
aeid_150	0.791	0.428	5
aeid_248	0.790	0.553	5

Endpoint	mean AUROC	mean AUPRC	folds
aeid_156	0.790	0.441	5
aeid_252	0.789	0.666	5
aeid_232	0.789	0.369	5
aeid_244	0.788	0.624	5
aeid_302	0.788	0.610	5
aeid_2893	0.787	0.656	5
aeid_172	0.787	0.675	5
aeid_158	0.786	0.723	5
aeid_206	0.785	0.494	5
aeid_2861	0.783	0.470	5
aeid_2887	0.782	0.470	5
aeid_2871	0.782	0.451	5
aeid_166	0.781	0.517	5
aeid_236	0.780	0.638	5
aeid_278	0.780	0.570	5
aeid_286	0.780	0.542	5
aeid_268	0.778	0.593	5
aeid_256	0.777	0.550	5
aeid_2857	0.775	0.395	4
aeid_2869	0.775	0.546	5
aeid_2825	0.775	0.611	5
aeid_2835	0.774	0.488	5
aeid_2815	0.774	0.537	5
aeid_2813	0.774	0.557	5
aeid_304	0.772	0.588	5
aeid_194	0.772	0.745	5
aeid_2863	0.770	0.468	5
aeid_2903	0.770	0.542	5
aeid_2925	0.769	0.428	5
aeid_2909	0.768	0.506	5
aeid_266	0.768	0.506	5

Endpoint	mean AUROC	mean AUPRC	folds
aeid_168	0.766	0.475	5
aeid_2837	0.766	0.465	5
aeid_316	0.765	0.607	5
aeid_234	0.765	0.602	5
aeid_240	0.763	0.509	5
aeid_306	0.762	0.506	5
aeid_146	0.761	0.542	5
aeid_154	0.760	0.554	5
aeid_198	0.759	0.457	5
aeid_188	0.758	0.586	5
aeid_2889	0.757	0.579	5
aeid_2811	0.757	0.507	5
aeid_3213	0.756	0.309	5
aeid_282	0.755	0.576	5
aeid_258	0.755	0.645	5
aeid_2841	0.754	0.469	5
aeid_148	0.754	0.526	5
aeid_246	0.753	0.618	5
aeid_202	0.751	0.552	5
aeid_3222	0.751	0.406	5
aeid_2901	0.750	0.624	5
aeid_180	0.747	0.513	5
aeid_300	0.747	0.534	5
aeid_294	0.745	0.559	5
aeid_228	0.745	0.537	5
aeid_2867	0.743	0.533	5
aeid_164	0.742	0.518	5
aeid_2883	0.740	0.633	5
aeid_2885	0.739	0.448	5
aeid_262	0.733	0.561	5
aeid_284	0.731	0.589	5

Endpoint	mean AUROC	mean AUPRC	folds
aeid_290	0.726	0.440	5
aeid_308	0.724	0.539	5
aeid_2927	0.719	0.618	5
aeid_242	0.719	0.563	5
aeid_2911	0.716	0.468	5
aeid_2923	0.716	0.370	5
aeid_214	0.714	0.465	5
aeid_2847	0.713	0.442	5
aeid_216	0.708	0.283	5
aeid_176	0.704	0.602	5
aeid_2849	0.704	0.598	5
aeid_2899	0.702	0.640	5
aeid_254	0.701	0.641	5
aeid_2933	0.701	0.537	5
aeid_208	0.699	0.423	5
aeid_314	0.697	0.530	5
aeid_230	0.696	0.441	5
aeid_2915	0.696	0.580	5
aeid_184	0.690	0.436	5
aeid_2859	0.689	0.487	5
aeid_2921	0.686	0.527	5
aeid_210	0.683	0.344	5
aeid_2839	0.676	0.521	5
aeid_2809	0.676	0.476	5
aeid_224	0.673	0.531	5
aeid_3216	0.671	0.201	5
aeid_2817	0.671	0.552	5
aeid_2931	0.670	0.598	5
aeid_238	0.669	0.443	5
aeid_220	0.668	0.476	5
aeid_152	0.663	0.466	5

Endpoint	mean AUROC	mean AUPRC	folds
aeid_222	0.658	0.516	5
aeid_212	0.654	0.332	5
aeid_3219	0.634	0.126	5
aeid_218	0.632	0.366	5
aeid_162	0.624	0.483	5
aeid_2823	0.606	0.679	5
aeid_3215	0.594	0.198	5
aeid_3214	0.593	0.294	5
aeid_75	0.518	0.099	3

S2. Leave-overlap-out chemistry split (soil-water K_d)

The 42 K_d PFAS partitioned into the 23 that share a structure with the 209 ToxCast PFAS (in-distribution) and the 19 whose chemistry is unseen there (held-out); the latter is the test set for the leave-overlap-out transfer result (Table S2).

Table S2: Leave-overlap-out split for the soil-water K_d transfer test (23 in-distribution PFAS that share a structure with the 209 ToxCast PFAS; 19 held-out PFAS whose chemistry is unseen there). The model is trained on the in-distribution set and tested on the held-out set.

PFAS	DTXSID	Split	SMILES
4:2 FTOH	DTXSID7060007	in-distribution	OCCC(F)(F)C(F)(F)C(F)(F)C(F)(F)F
4:2 FTS	DTXSID4067331	in-distribution	OS(=O)(=O)CCC(F)(F)C(F)(F)C(F)(F)C(F)(F)F
6:2 FTOH	DTXSID5044572	in-distribution	OCCC(F)(F)C(F)(F)C(F)(F)C(F)(F)C(F)(F)C(F)(F)F
6:2 FTS	DTXSID5048220	in-distribution	OS(=O)(=O)CCC(F)(F)C(F)(F)C(F)(F)C(F)(F)C(F)(F)C(F)(F)F

PFAS	DTXSID	Split	SMILES
8:2 FTOH	DTXSID7029904	in-distribution	OCCC(F)(F)C(F)(F)C(F)(F)C(F)(F)C(F)(F)C(F)(F)C(F)(F)C(F)(F)F
8:2 FTS	DTXSID3057898	in-distribution	OS(=O)(=O)CCC(F)(F)C(F)(F)C(F)(F)C(F)(F)C(F)(F)C(F)(F)C(F)(F)C(F)(F)F
EtFOSA	DTXSID1032646	in-distribution	CCNS(=O)(=O)C(F)(F)C(F)(F)C(F)(F)C(F)(F)C(F)(F)C(F)(F)C(F)(F)C(F)(F)F
FHxSA	DTXSID00872222	in-distribution	NS(=O)(=O)C(F)(F)C(F)(F)C(F)(F)C(F)(F)C(F)(F)C(F)(F)F
GenX	DTXSID70880215	in-distribution	OC(=O)C(F)(C(F)(F)F)OC(F)(F)C(F)(F)C(F)(F)F
PFBA	DTXSID4059916	in-distribution	OC(=O)C(F)(F)C(F)(F)C(F)(F)F
PFBS	DTXSID5030030	in-distribution	OS(=O)(=O)C(F)(F)C(F)(F)C(F)(F)C(F)(F)F
PFDA	DTXSID3031860	in-distribution	OC(=O)C(F)(F)C(F)(F)C(F)(F)C(F)(F)C(F)(F)C(F)(F)C(F)(F)C(F)(F)C(F)(F)F
PFHpA	DTXSID1037303	in-distribution	OC(=O)C(F)(F)C(F)(F)C(F)(F)C(F)(F)C(F)(F)C(F)(F)F
PFHpS	DTXSID8059921	in-distribution	OS(=O)(=O)C(F)(F)C(F)(F)C(F)(F)C(F)(F)C(F)(F)C(F)(F)C(F)(F)F
PFHxA	DTXSID3031862	in-distribution	OC(=O)C(F)(F)C(F)(F)C(F)(F)C(F)(F)C(F)(F)F
PFHxS	DTXSID7040150	in-distribution	OS(=O)(=O)C(F)(F)C(F)(F)C(F)(F)C(F)(F)C(F)(F)C(F)(F)F

PFAS	DTXSID	Split	SMILES
PFNA	DTXSID8031863	in-distribution	OC(=O)C(F)(F)C(F)(F)C(F)(F)C(F)(F)C(F)(F)C(F)(F)C(F)(F)C(F)(F)F
PFOA	DTXSID8031865	in-distribution	OC(=O)C(F)(F)C(F)(F)C(F)(F)C(F)(F)C(F)(F)C(F)(F)C(F)(F)F
PFOS	DTXSID3031864	in-distribution	OS(=O)(=O)C(F)(F)C(F)(F)C(F)(F)C(F)(F)C(F)(F)C(F)(F)C(F)(F)C(F)(F)F
PFOSA	DTXSID3038939	in-distribution	NS(=O)(=O)C(F)(F)C(F)(F)C(F)(F)C(F)(F)C(F)(F)C(F)(F)C(F)(F)C(F)(F)F
PFPeA	DTXSID6062599	in-distribution	OC(=O)C(F)(F)C(F)(F)C(F)(F)C(F)(F)F
PFPeS	DTXSID8059920	in-distribution	OS(=O)(=O)C(F)(F)C(F)(F)C(F)(F)C(F)(F)C(F)(F)F
PFUnA	DTXSID8047553	in-distribution	OC(=O)C(F)(F)C(F)(F)C(F)(F)C(F)(F)C(F)(F)C(F)(F)C(F)(F)C(F)(F)C(F)(F)C(F)(F)F
10:2 FTOH	DTXSID3047579	**held-out**	OCCC(F)(F)C(F)(F)C(F)(F)C(F)(F)C(F)(F)C(F)(F)C(F)(F)C(F)(F)C(F)(F)C(F)(F)F
10:2 FtSaB	DTXSID90880224	**held-out**	C[N+](C)(CCCNS(=O)(=O)CCC(F)(F)C(F)(F)C(F)(F)C(F)(F)C(F)(F)C(F)(F)C(F)(F)C(F)(F)C(F)(F)C(F)(F)F)CC(=O)[O-]
6:2 FtSaB	DTXSID70880222	**held-out**	C[N+](C)(CCCNS(=O)(=O)CCC(F)(F)C(F)(F)C(F)(F)C(F)(F)C(F)(F)C(F)(F)F)CC(=O)[O-]

PFAS	DTXSID	Split	SMILES
8:2 Cl-PFAES	DTXSID40892109	**held-out**	ClC(F)(F)C(F)(F)C(F)(F)C(F)(F)OC(F)(F)C(F)(F)C(F)(F)C(F)(F)S(=O)(=O)O
8:2 FtSaB	DTXSID80880223	**held-out**	C[N+](C)(CCCNS(=O)(=O)CCC(F)(F)C(F)(F)C(F)(F)C(F)(F)C(F)(F)C(F)(F)C(F)(F)C(F)(F)F)CC(=O)[O-]
ADONA	DTXSID90892109	**held-out**	OC(=O)CC(F)(F)OCC(F)(F)C(F)(F)C(F)(F)OCC(F)(F)F
AmPr-FHxSA	DTXSID40872212	**held-out**	NCCCNS(=O)(=O)C(F)(F)C(F)(F)C(F)(F)C(F)(F)C(F)(F)C(F)(F)F
FBSA	DTXSID8047572	**held-out**	NS(=O)(=O)C(F)(F)C(F)(F)C(F)(F)C(F)(F)F
PFDPA	DTXSID40892132	**held-out**	OP(=O)(O)C(F)(F)C(F)(F)C(F)(F)C(F)(F)C(F)(F)C(F)(F)C(F)(F)C(F)(F)C(F)(F)C(F)(F)F
PFDS	DTXSID8059923	**held-out**	OS(=O)(=O)C(F)(F)C(F)(F)C(F)(F)C(F)(F)C(F)(F)C(F)(F)C(F)(F)C(F)(F)C(F)(F)C(F)(F)F
PFDoA	DTXSID8031077	**held-out**	OC(=O)C(F)(F)C(F)(F)C(F)(F)C(F)(F)C(F)(F)C(F)(F)C(F)(F)C(F)(F)C(F)(F)C(F)(F)C(F)(F)F
PFEtCHxS	DTXSID60872214	**held-out**	OS(=O)(=O)C(F)(F)C(F)(F)CC1(F)C(F)(F)C(F)(F)C(F)(F)C(F)(F)C1(F)F
PFHxPA	DTXSID40892130	**held-out**	OP(=O)(O)C(F)(F)C(F)(F)C(F)(F)C(F)(F)C(F)(F)C(F)(F)F

PFAS	DTXSID	Split	SMILES
PFNS	DTXSID8059922	**held-out**	OS(=O)(=O)C(F)(F)C(F)(F)C(F)(F)C(F)(F)C(F)(F)C(F)(F)C(F)(F)C(F)(F)C(F)(F)F
PFOAAmS	DTXSID30872211	**held-out**	OC(=O)CCNS(=O)(=O)C(F)(F)C(F)(F)C(F)(F)C(F)(F)C(F)(F)C(F)(F)C(F)(F)C(F)(F)F
PFOAB	DTXSID20872210	**held-out**	C[N+](C)(CCCNS(=O)(=O)C(F)(F)C(F)(F)C(F)(F)C(F)(F)C(F)(F)C(F)(F)C(F)(F)C(F)(F)F)CC(=O)[O-]
PFOPA	DTXSID40892131	**held-out**	OP(=O)(O)C(F)(F)C(F)(F)C(F)(F)C(F)(F)C(F)(F)C(F)(F)C(F)(F)C(F)(F)F
TAmPr-FHxSA	DTXSID50872213	**held-out**	C[N+](C)(C)CCCNS(=O)(=O)C(F)(F)C(F)(F)C(F)(F)C(F)(F)C(F)(F)C(F)(F)F
TFA	DTXSID1027302	**held-out**	OC(=O)C(F)(F)F

S3. Subfamily-out (true OOD) results

Per-subfamily performance under leave-one-subfamily-out cross-validation, alongside the in-distribution per-subfamily values (Table S3).

Table S3: Leave-one-subfamily-out (true OOD) results: each subfamily is held out entirely and the matched multi-task MolFormer is fine-tuned on the rest. In-distribution AUROC is the per-subfamily value under generic Butina CV (main-text Table 8). PFESA ($n = 1$) is unevaluable and omitted.

Subfamily	n	eval. endpts	in-dist AUROC	OOD AUROC	OOD AUPRC
FTOH	15	137	0.787	0.816	0.744

Subfamily	n	eval. endpts	in-dist AUROC	OOD AUROC	OOD AUPRC
PFCA	25	152	0.777	0.794	0.550
PFECA	7	145	0.906	0.791	0.619
PFSA	11	111	0.724	0.764	0.624
OTHER	49	147	0.690	0.728	0.443
UVCB_PROFLUO	93	156	0.710	0.588	0.289
FOSA_FOSE	8	146	0.427	0.412	0.607

S4. PFAS subfamily assignments

Rule-based subfamily label and canonical SMILES for each of the 209 evaluation PFAS (Table S4).

Table S4: PFAS subfamily assignments for the 209 evaluation compounds (rule-based classifier, `pfas_b.subfamilies`). Used for the subfamily-out CV (Table S3).

DTXSID	Subfamily	SMILES
DTXSID1032646	FOSA_FOSE	CCNS(=O)(=O)C(F)(F)C(F)(F)C(F)(F)C(F)(F)C(F)(F)C(F)(F)C(F)(F)C(F)(F)F
DTXSID1067629	FOSA_FOSE	CNS(=O)(=O)C(F)(F)C(F)(F)C(F)(F)C(F)(F)C(F)(F)C(F)(F)C(F)(F)C(F)(F)F
DTXSID3038939	FOSA_FOSE	NS(=O)(=O)C(F)(F)C(F)(F)C(F)(F)C(F)(F)C(F)(F)C(F)(F)C(F)(F)C(F)(F)F
DTXSID4041284	FOSA_FOSE	C[N+](C)(CCCNS(=O)(=O)CCC(F)(F)C(F)(F)C(F)(F)C(F)(F)C(F)(F)C(F)(F)F)CC([O-])=O

DTXSID	Subfamily	SMILES
DTXSID50469320	FOSA_FOSE	NS(=O)(=O)C(F)(F)C(F)(F)C(F)(F)C(F)(F)C(F)(F)C(F)(F)F
DTXSID6027426	FOSA_FOSE	CCN(CCO)S(=O)(=O)C(F)(F)C(F)(F)C(F)(F)C(F)(F)C(F)(F)C(F)(F)C(F)(F)C(F)(F)F
DTXSID7027831	FOSA_FOSE	CN(CCO)S(=O)(=O)C(F)(F)C(F)(F)C(F)(F)C(F)(F)C(F)(F)C(F)(F)C(F)(F)C(F)(F)F
DTXSID8051419	FOSA_FOSE	[I-].C[N+](C)(C)CCCNS(=O)(=O)C(F)(F)C(F)(F)C(F)(F)C(F)(F)C(F)(F)C(F)(F)C(F)(F)C(F)(F)F
DTXSID00194615	FTOH	FC(F)C(F)(F)C(F)(F)C(F)(F)C(F)(F)C(F)(F)C(F)(F)C(F)(F)COC(=O)C=C
DTXSID0059879	FTOH	OCC(F)(F)C(F)(F)C(F)(F)C(F)F
DTXSID10379850	FTOH	FC(F)C(F)(F)C(F)(F)C(F)(F)C(F)=C(F)F
DTXSID20337466	FTOH	FC(F)C(F)(F)C(F)(F)C(F)(F)C=O
DTXSID30340244	FTOH	CC1=CC=C(C=C1)S(=O)(=O)OCC(F)(F)C(F)(F)C(F)(F)C(F)(F)C(F)(F)C(F)F
DTXSID50226894	FTOH	OC(=O)C(F)(F)C(F)(F)C(F)(F)C(F)(F)C(F)(F)C(F)(F)C(F)(F)C(F)F
DTXSID5061954	FTOH	OC(=O)C(F)(F)C(F)(F)C(F)(F)C(F)(F)C(F)(F)C(F)(F)C(F)(F)C(F)(F)C(F)(F)C(F)F
DTXSID50880218	FTOH	FC1CC(F)(F)C(F)(F)C1(F)F
DTXSID50892417	FTOH	OC(=O)C(F)(F)C(F)(F)C(F)F
DTXSID70366226	FTOH	NC(=O)C(F)(F)C(F)(F)C(F)(F)C(F)F
DTXSID70379730	FTOH	FC(F)C(F)(F)C(F)(F)C(F)(F)C(F)=O
DTXSID70565479	FTOH	OC(=O)C(F)(F)C(F)(F)C(F)(F)C(F)(F)C(F)(F)C(F)(F)C(F)F

DTXSID	Subfamily	SMILES
DTXSID8052017	FTOH	FC(OC(F)(F)C(F)(F)C(F)(F)F)C(F)(F)F
DTXSID9059832	FTOH	OCC(F)(F)C(F)(F)C(F)(F)C(F)(F)C(F)(F)C(F)F
DTXSID90880131	FTOH	CC(=C)C(=O)OCC(F)(F)C(F)(F)C(F)(F)C(F)F
DTXSID00379884	OTHER	FC(F)(F)C(F)(C(F)(F)F)C(F)(F)C(F)(F)CC1CO1
DTXSID00380798	OTHER	OCC(F)(F)OC(F)(F)C(F)(F)OC(F)(F)C(F)(F)OC(F)(F)CO
DTXSID00442840	OTHER	FC(F)(F)OCCOS(=O)(=O)C(F)(F)F
DTXSID0059871	OTHER	NC(=O)C(F)(F)C(F)(F)F
DTXSID0074059	OTHER	FC(F)OCC(F)(F)C(F)(F)F
DTXSID10224331	OTHER	FC(F)C(F)(F)COC(=O)C=C
DTXSID10370988	OTHER	FC(F)(F)C(F)(OCC=C)C(F)(F)F
DTXSID20880144	OTHER	CCOC(=O)CC(=O)C(F)(F)C(F)(F)F
DTXSID20881338	OTHER	COC(F)(C(F)(F)C(F)(F)F)C(F)(C(F)(F)F)C(F)(F)F
DTXSID30170109	OTHER	OC(=O)C=C(C(F)(F)F)C(F)(F)F
DTXSID3032620	OTHER	FC(F)C(F)(F)OC1=C(Cl)C=C(NC(=O)NC(=O)C2=C(F)C=CC=C2F)C=C1Cl
DTXSID30378880	OTHER	FC(F)COS(=O)(=O)C(F)(F)F
DTXSID30395037	OTHER	FC(F)(F)COC(OCC(F)(F)F)OCC(F)(F)F
DTXSID3047136	OTHER	FC1=C(F)C(F)=C(Br)C(F)=C1F
DTXSID30622698	OTHER	FC(=C)C(=O)OC(C(F)(F)F)C(F)(F)F
DTXSID4022369	OTHER	[H][C@@]12CC[C@H](O)[C@@]1(C)CC[C@]1([H])C3=C(C[C@@H](CCCCCCCCCS(=O)CCCC(F)(F)C(F)(F)F)[C@@]21[H])C=C(O)C=C3

DTXSID	Subfamily	SMILES
DTXSID40380257	OTHER	OC(=O)C=CC(F)(C(F)(F)F)C(F)(F)F
DTXSID40380797	OTHER	OCC(F)(F)OC(F)(F)C(F)(F)OC(F)(F)C(F)(F)OC(F)(F)F
DTXSID4047672	OTHER	CC1=CC(=CC=C1NC(=O)C1=CC=CC(I)=C1C(=O)NC(C)(C)CS(C)(=O)=O)C(F)(C(F)(F)F)C(F)(F)F
DTXSID4061753	OTHER	FC(F)(F)C(=O)CC(=O)C(F)(F)F
DTXSID5034357	OTHER	FC(C(F)(F)F)C(F)(F)OC1=CC(Cl)=C(NC(=O)NC(=O)C2=C(F)C=CC=C2F)C=C1Cl
DTXSID50379359	OTHER	FC(F)(F)C(F)(F)C(F)(C=C)C(F)(F)F
DTXSID50381992	OTHER	FC(F)(F)C(F)(F)CNCC(F)(F)C(F)(F)F
DTXSID5042326	OTHER	COC(F)(F)C(F)(C(F)(F)F)C(F)(F)F
DTXSID5046516	OTHER	FC(F)(F)COCC(F)(F)F
DTXSID5047328	OTHER	O.[K+].NC(=O)C1=NC(=N[N-]1)C1=CC=CC(=C1)C1=CC(F)=CC=C1OCC(F)(F)C(F)(F)F
DTXSID5073901	OTHER	CC(F)(F)CC(F)(F)F
DTXSID60238701	OTHER	OC(C(O)(C(F)(F)F)C(F)(F)F)(C(F)(F)F)C(F)(F)F
DTXSID6061022	OTHER	FC1(F)C(=O)OC(=O)C1(F)F
DTXSID7037717	OTHER	OC1=CC=C(C=C1)C(C1=CC=C(O)C=C1)(C(F)(F)F)C(F)(F)F
DTXSID70379295	OTHER	OC(O)(C(F)C(O)(O)C(F)(F)F)C(F)(F)F
DTXSID70381090	OTHER	OCC(F)(F)OC(F)(F)C(F)(F)OC(F)(F)CO
DTXSID70481246	OTHER	NC(C(F)(F)F)C(F)(F)F
DTXSID70537191	OTHER	COC(F)(C(F)F)C(F)(F)F

DTXSID	Subfamily	SMILES
DTXSID7059933	OTHER	FC(F)(F)N1C(F)(F)C(F)(F)OC(F)(F)C1(F)F
DTXSID70870515	OTHER	FC(F)(F)C(F)(F)C(=O)OC(=O)C(F)(F)C(F)(F)F
DTXSID70880134	OTHER	CC(O)C(F)(F)C(F)(F)F
DTXSID70880230	OTHER	FC(F)C(F)(F)COCC1CO1
DTXSID8034956	OTHER	FC(F)C(F)(F)OCC(CN1C=NC=N1)C1=C(Cl)C=C(Cl)C=C1
DTXSID80380256	OTHER	OC(=O)CCC(F)(C(F)(F)F)C(F)(F)F
DTXSID80382093	OTHER	NC(O)(C(F)(F)F)C(F)(F)F
DTXSID8044969	OTHER	COC(=O)C(F)(F)C(F)(F)OC(F)(C(F)(F)F)C(F)(F)OC(F)=C(F)F
DTXSID8046614	OTHER	FCOC(C(F)(F)F)C(F)(F)F
DTXSID8059928	OTHER	OC(=O)C(F)(F)C(F)(F)C(O)=O
DTXSID8059970	OTHER	OC(=O)C(F)(F)C(F)(F)F
DTXSID90174941	OTHER	FC(F)(F)C(=O)CC(=O)C(F)(F)C(F)(F)F
DTXSID90275806	OTHER	OC(=CC(=O)C(F)(F)F)C(F)(F)F
DTXSID90285748	OTHER	CC(=O)C(F)(F)C(F)(F)F
DTXSID9059969	OTHER	OC(O)C(F)(F)C(F)(F)F
DTXSID00379268	PFCA	OC(=O)CCC(F)(F)C(F)(F)C(F)(F)F
DTXSID00880026	PFCA	[K+].[O-]C(=O)C(F)(F)C(F)(F)C(F)(F)C(F)(F)C(F)(F)C(F)(F)C(F)(F)F
DTXSID00892442	PFCA	OC(=O)C(F)(OC(F)(F)C(F)(OC(F)(F)C(F)(F)C(F)(F)F)C(F)(F)F)C(F)(F)F
DTXSID1037303	PFCA	OC(=O)C(F)(F)C(F)(F)C(F)(F)C(F)(F)C(F)(F)C(F)(F)F
DTXSID20874028	PFCA	OC(=O)CCC(F)(F)C(F)(F)C(F)(F)C(F)(F)C(F)(F)F

DTXSID	Subfamily	SMILES
DTXSID3031860	PFCA	OC(=O)C(F)(F)C(F)(F)C(F)(F)C(F)(F)C(F)(F)C(F)(F)C(F)(F)C(F)(F)C(F)(F)F
DTXSID3031862	PFCA	OC(=O)C(F)(F)C(F)(F)C(F)(F)C(F)(F)C(F)(F)F
DTXSID30382104	PFCA	OC(=O)C(F)(F)C(F)(F)C(F)(F)C(F)(F)C(F)(F)C(F)(F)C(F)(F)C(F)(F)Cl
DTXSID3059921	PFCA	OC(=O)C(F)(F)C(F)(F)C(F)(F)C(F)(F)C(F)(F)C(F)(F)C(F)(F)C(F)(F)C(F)(F)C(F)(F)C(F)(F)C(F)(F)C(F)(F)F
DTXSID40108559	PFCA	[NH4+].[O-]C(=O)C(F)(OC(F)(F)C(F)(F)C(F)(F)F)C(F)(F)F
DTXSID4059833	PFCA	OC(=O)C(F)(F)C(F)(F)C(F)(F)C(F)(F)C(O)=O
DTXSID4059916	PFCA	OC(=O)C(F)(F)C(F)(F)C(F)(F)F
DTXSID40880025	PFCA	[Na+].[O-]C(=O)C(F)(F)C(F)(F)C(F)(F)C(F)(F)C(F)(F)C(F)(F)C(F)(F)F
DTXSID6062599	PFCA	OC(=O)C(F)(F)C(F)(F)C(F)(F)C(F)(F)F
DTXSID60663110	PFCA	OC(=O)C(F)(F)C(F)(F)C(F)(F)OC(F)(C(F)(F)F)C(F)(F)F
DTXSID70276659	PFCA	OC(=O)C(F)(OC(F)(F)C(F)(OC(F)(F)C(F)(OC(F)(F)C(F)(F)C(F)(F)F)C(F)(F)F)C(F)(F)F)C(F)(F)F
DTXSID70379917	PFCA	OC(=O)CCC(F)(F)C(F)(F)C(F)(F)C(F)(F)C(F)(F)C(F)(F)F
DTXSID70880215	PFCA	OC(=O)C(F)(OC(F)(F)C(F)(F)C(F)(F)F)C(F)(F)F
DTXSID8031863	PFCA	OC(=O)C(F)(F)C(F)(F)C(F)(F)C(F)(F)C(F)(F)C(F)(F)C(F)(F)C(F)(F)F

DTXSID	Subfamily	SMILES
DTXSID8031865	PFCA	OC(=O)C(F)(F)C(F)(F)C(F)(F)C(F)(F)C(F)(F)C(F)(F)C(F)(F)F
DTXSID8037708	PFCA	[NH4+].[O-]C(=O)C(F)(F)C(F)(F)C(F)(F)C(F)(F)C(F)(F)C(F)(F)C(F)(F)F
DTXSID8047553	PFCA	OC(=O)C(F)(F)C(F)(F)C(F)(F)C(F)(F)C(F)(F)C(F)(F)C(F)(F)C(F)(F)C(F)(F)C(F)(F)F
DTXSID8059926	PFCA	OC(=O)C(F)(F)C(F)(F)C(F)(F)C(O)=O
DTXSID90382620	PFCA	OC(=O)CCC(F)(F)C(F)(F)C(F)(F)C(F)(F)C(F)(F)C(F)(F)F
DTXSID90868151	PFCA	OC(=O)C(F)(F)C(F)(F)C(F)(F)C(F)(F)C(F)(F)C(F)(F)C(F)(F)C(F)(F)C(F)(F)C(F)(F)C(F)(F)F
DTXSID20375106	PFECA	OC(=O)C(F)(F)OC(F)(F)C(F)(F)OC(F)(F)C(O)=O
DTXSID30382063	PFECA	OC(=O)C(F)(F)OC(F)(F)C(F)(F)OC(F)(F)F
DTXSID50375114	PFECA	OC(=O)C(F)(F)OC(F)(F)C(F)(F)OC(F)(F)C(F)(F)OC(F)(F)C(F)(F)C(F)(F)C(F)(F)F
DTXSID50381073	PFECA	OC(=O)C(F)(F)OC(F)(F)C(F)(F)OC(F)(F)C(F)(F)C(F)(F)C(F)(F)F
DTXSID60500450	PFECA	OC(=O)C(F)(F)C(F)(F)C(F)(F)OC(F)(F)F
DTXSID70191136	PFECA	OC(=O)C(F)(F)C(F)(F)OC(F)(F)F
DTXSID80380837	PFECA	OC(=O)C(F)(F)OC(F)(F)C(F)(F)OC(F)(F)C(F)(F)OC(F)(F)F
DTXSID50379814	PFESA	OS(=O)(=O)C(F)(F)C(F)(F)OC(F)(F)C(F)(F)F

DTXSID	Subfamily	SMILES
DTXSID00192353	PFSA	OS(=O)(=O)CCC(F)(F)C(F)(F)C(F)(F)C(F)(F)C(F)(F)C(F)(F)C(F)(F)C(F)(F)F
DTXSID3031864	PFSA	OS(=O)(=O)C(F)(F)C(F)(F)C(F)(F)C(F)(F)C(F)(F)C(F)(F)C(F)(F)C(F)(F)F
DTXSID3037707	PFSA	[K+].[O-]S(=O)(=O)C(F)(F)C(F)(F)C(F)(F)C(F)(F)F
DTXSID3037709	PFSA	[K+].[O-]S(=O)(=O)C(F)(F)C(F)(F)C(F)(F)C(F)(F)C(F)(F)C(F)(F)F
DTXSID30891564	PFSA	OS(=O)(=O)CCC(F)(F)C(F)(F)C(F)(F)C(F)(F)F
DTXSID5030030	PFSA	OS(=O)(=O)C(F)(F)C(F)(F)C(F)(F)C(F)(F)F
DTXSID6067331	PFSA	OS(=O)(=O)CCC(F)(F)C(F)(F)C(F)(F)C(F)(F)C(F)(F)C(F)(F)F
DTXSID60892443	PFSA	[Na+].[O-]S(=O)(=O)C(F)(F)C(F)(F)C(F)(F)C(F)(F)C(F)(F)C(F)(F)C(F)(F)C(F)(F)C(F)(F)C(F)(F)F
DTXSID7040150	PFSA	OS(=O)(=O)C(F)(F)C(F)(F)C(F)(F)C(F)(F)C(F)(F)C(F)(F)F
DTXSID8037706	PFSA	[K+].[O-]S(=O)(=O)C(F)(F)C(F)(F)C(F)(F)C(F)(F)C(F)(F)C(F)(F)C(F)(F)C(F)(F)F
DTXSID8059920	PFSA	OS(=O)(=O)C(F)(F)C(F)(F)C(F)(F)C(F)(F)C(F)(F)C(F)(F)C(F)(F)F
DTXSID00190950	UVCB_PROFLUO	OCC(F)(F)C(F)(F)C(F)(F)C(F)(F)C(F)(F)C(F)(F)F
DTXSID00223797	UVCB_PROFLUO	C[Si](Cl)(Cl)CCC(F)(F)C(F)(F)C(F)(F)C(F)(F)C(F)(F)C(F)(F)F

DTXSID	Subfamily	SMILES
DTXSID0036926	UVCB_PROFLUO	FC(F)(F)C1(F)C(F)(F)C(F)(F)C(F)(F)C(F)(C(F)(F)F)C1(F)F
DTXSID00379925	UVCB_PROFLUO	FC(F)(F)C(F)(F)C(F)(F)C(F)(F)C(F)(F)C(F)(F)C(F)(F)C(F)(F)C(Cl)=O
DTXSID0047583	UVCB_PROFLUO	FC(F)(F)C(F)(F)C(F)(F)C(F)(F)I
DTXSID0059794	UVCB_PROFLUO	FC(F)(F)C(F)(F)C(F)(F)C(F)(F)C(F)(F)C(F)(F)C(F)(F)C(F)(F)F
DTXSID0059829	UVCB_PROFLUO	FC(=O)C(F)(F)C(F)(F)C(F)(F)C(F)(F)C(F)(F)C(F)(F)C(F)(F)F
DTXSID0060985	UVCB_PROFLUO	FC(F)(C(Cl)=O)C(F)(F)C(F)(F)C(Cl)=O
DTXSID0061826	UVCB_PROFLUO	FC(F)=C(F)OC(F)(F)C(F)(F)C(F)(F)F
DTXSID0073118	UVCB_PROFLUO	CCOC(F)(F)C(F)(F)C(F)(F)C(F)(F)F
DTXSID10190946	UVCB_PROFLUO	FC(F)(F)C(F)(F)C(F)(F)C=O
DTXSID10379254	UVCB_PROFLUO	FC(F)(F)C(F)(F)C(F)(F)CC1CO1
DTXSID10379835	UVCB_PROFLUO	NCC(F)(F)C(F)(F)C(F)(F)C(F)(F)C(F)(F)C(F)(F)F
DTXSID10379991	UVCB_PROFLUO	OCCCC(F)(F)C(F)(F)C(F)(F)C(F)(F)C(F)(F)C(F)(F)C(F)(F)C(F)(F)F
DTXSID10382147	UVCB_PROFLUO	OCC(O)CC(F)(F)C(F)(F)C(F)(F)C(F)(F)F
DTXSID10461880	UVCB_PROFLUO	FC1(F)C=CC(F)(F)C1(F)F
DTXSID1047029	UVCB_PROFLUO	FC1(F)C(F)(F)C(F)(F)C2(F)C(F)(C1(F)F)C(F)(F)C(F)(F)C1(F)C(F)(F)C(F)(F)C(F)(F)C(F)(F)C21F
DTXSID1047578	UVCB_PROFLUO	FC(F)(F)C(F)(F)C(F)(F)C(F)(F)CCI
DTXSID1062122	UVCB_PROFLUO	OCCC(F)(F)C(F)(F)C(F)(F)C(F)(F)F
DTXSID1068772	UVCB_PROFLUO	FC(F)(F)C(F)(F)C(F)(F)C(F)(F)CCOC(=O)C=C

DTXSID	Subfamily	SMILES
DTXSID1074915	UVCB_PROFLUO	CCO[Si](CCC(F)(F)C(F)(F)C(F)(F)C(F)(F)C(F)(F)C(F)(F)F)(OCC)OCC
DTXSID20179883	UVCB_PROFLUO	OP(O)(=O)CCC(F)(F)C(F)(F)C(F)(F)C(F)(F)C(F)(F)C(F)(F)F
DTXSID20335129	UVCB_PROFLUO	FC(F)(Br)C(F)(F)C(F)(F)C(F)(F)C(F)(F)C(F)(F)Br
DTXSID20335700	UVCB_PROFLUO	COC(=O)C(F)(F)C(F)(F)C(F)(F)C(F)(F)C(F)(F)F
DTXSID20337446	UVCB_PROFLUO	FC(F)(F)C(F)(F)C(F)(F)C(F)(F)C(F)(F)C(F)(F)C(F)(F)C(F)(F)CCS
DTXSID20379947	UVCB_PROFLUO	FC(F)(F)C(F)(F)C(F)(F)C(F)(F)C(F)(F)C(F)(F)CCS
DTXSID20440585	UVCB_PROFLUO	CCCCCCCCC(F)(F)C(F)(F)C(F)(F)C(F)(F)C(F)(F)C(F)(F)F
DTXSID2047565	UVCB_PROFLUO	FC(F)(F)C(F)(F)C(F)(F)C(F)(F)C(F)(F)C(F)(F)CCI
DTXSID2060965	UVCB_PROFLUO	NC(=O)C(F)(F)C(F)(F)C(F)(F)F
DTXSID20861913	UVCB_PROFLUO	FC(F)(F)C(F)(F)C(F)(F)C(F)(F)S(F)(=O)=O
DTXSID30190948	UVCB_PROFLUO	FC(F)(I)C(F)(F)C(F)(F)C(F)(F)I
DTXSID30396867	UVCB_PROFLUO	OCC(F)(F)C(F)(F)C(F)(F)C(F)(F)C(F)(F)C(F)(F)CO
DTXSID3047558	UVCB_PROFLUO	CC(=C)C(=O)OCCC(F)(F)C(F)(F)C(F)(F)C(F)(F)C(F)(F)C(F)(F)F
DTXSID3059927	UVCB_PROFLUO	OCC(F)(F)C(F)(F)C(F)(F)CO
DTXSID3059971	UVCB_PROFLUO	FC(F)(F)C(F)(F)C(F)(F)Br
DTXSID30627108	UVCB_PROFLUO	OP(O)(=O)CCC(F)(F)C(F)(F)C(F)(F)C(F)(F)C(F)(F)C(F)(F)C(F)(F)C(F)(F)F
DTXSID3065586	UVCB_PROFLUO	CC(=C)C(=O)OCC(F)(F)C(F)(F)C(F)(F)F

DTXSID	Subfamily	SMILES
DTXSID3066215	UVCB_PROFLUO	CC(C)(C)C(=O)CC(=O)C(F)(F)C(F)(F)C(F)(F)F
DTXSID30880413	UVCB_PROFLUO	FC(F)(F)C(F)(F)C(F)(F)C(F)(F)C(F)(F)C(F)(F)CC1CO1
DTXSID40187142	UVCB_PROFLUO	FC(F)(F)C(F)(F)C(F)(F)C(F)(F)C(F)(F)C(F)(F)C(F)(F)C(Cl)=O
DTXSID40204489	UVCB_PROFLUO	FC(F)=C(F)C(F)(F)C(F)(F)C(F)(F)C(F)(F)C(F)(F)C(F)(F)F
DTXSID40235137	UVCB_PROFLUO	CO[Si](C)(CCC(F)(F)C(F)(F)C(F)(F)C(F)(F)C(F)(F)C(F)(F)F)OC
DTXSID40379666	UVCB_PROFLUO	CN(C(=O)C(F)(F)C(F)(F)C(F)(F)F)[Si](C)(C)C
DTXSID4059881	UVCB_PROFLUO	COC(=O)C(F)(F)C(F)(F)C(F)(F)F
DTXSID4059912	UVCB_PROFLUO	FC(F)(F)C(F)(F)C(F)(F)CI
DTXSID4059914	UVCB_PROFLUO	OCC(F)(F)C(F)(F)C(F)(F)F
DTXSID50184723	UVCB_PROFLUO	NCC(F)(F)C(F)(F)C(F)(F)C(F)(F)C(F)(F)C(F)(F)C(F)(F)F
DTXSID50218052	UVCB_PROFLUO	FC(=O)C(F)(F)C(F)(F)C(F)(F)C(F)=O
DTXSID50229163	UVCB_PROFLUO	FC(F)(F)C(F)(F)C(F)(F)C(F)(F)C(F)(F)C(F)(F)CC[Si](Cl)(Cl)Cl
DTXSID5027140	UVCB_PROFLUO	FC(F)(F)C(F)(F)C(F)(F)C(F)(F)C(F)(F)C(F)(F)C(F)(F)C(F)(F)S(F)(=O)=O
DTXSID50369896	UVCB_PROFLUO	OCC(F)(F)C(F)(F)C(F)(F)C(F)(F)C(F)(F)C(F)(F)C(F)(F)CO
DTXSID50379930	UVCB_PROFLUO	NCC(F)(F)C(F)(F)C(F)(F)C(F)(F)C(F)(F)C(F)(F)C(F)(F)C(F)(F)F
DTXSID50382621	UVCB_PROFLUO	OCCCC(F)(F)C(F)(F)C(F)(F)C(F)(F)C(F)(F)C(F)(F)F

DTXSID	Subfamily	SMILES
DTXSID50441560	UVCB_PROFLUO	COC(=O)CCC(F)(F)C(F)(F)C(F)(F)C(F)(F)F
DTXSID5044572	UVCB_PROFLUO	OCCC(F)(F)C(F)(F)C(F)(F)C(F)(F)C(F)(F)C(F)(F)F
DTXSID5059799	UVCB_PROFLUO	FC(F)(F)C(F)(F)C(F)(F)C(F)(F)C(F)(F)C(F)(F)C(F)(F)COC(=O)C=C
DTXSID5059828	UVCB_PROFLUO	FC(F)(F)C(F)(F)C(F)(F)C(F)(F)C(F)(F)C(F)(F)C(F)(F)I
DTXSID5060986	UVCB_PROFLUO	FC(F)(COC(=O)C=C)C(F)(F)C(F)(F)COC(=O)C=C
DTXSID5063235	UVCB_PROFLUO	CC(=C)C(=O)OCC(F)(F)C(F)(F)C(F)(F)C(F)(F)C(F)(F)C(F)(F)C(F)(F)F
DTXSID5067348	UVCB_PROFLUO	FC(F)(F)C(F)(F)C(F)(F)C(F)(F)C(F)(F)C(F)(F)C(F)(F)C(F)(F)CCOC(=O)C=C
DTXSID60195123	UVCB_PROFLUO	NC(=O)C(F)(F)C(F)(F)C(F)(F)C(F)(F)C(F)(F)C(F)(F)C(F)(F)F
DTXSID60377821	UVCB_PROFLUO	OCCCCC(F)(F)C(F)(F)C(F)(F)C(F)(F)F
DTXSID60377826	UVCB_PROFLUO	NCC(F)(F)C(F)(F)C(F)(F)C(F)(F)F
DTXSID60379269	UVCB_PROFLUO	OCCCC(F)(F)C(F)(F)C(F)(F)F
DTXSID60379901	UVCB_PROFLUO	CC(=C)C(=O)OCCC(F)(F)C(F)(F)C(F)(F)C(F)(F)C(F)(C(F)(F)F)C(F)(F)F
DTXSID60380390	UVCB_PROFLUO	FC(F)(F)COS(=O)(=O)C(F)(F)C(F)(F)C(F)(F)C(F)(F)F
DTXSID60400587	UVCB_PROFLUO	NC(=O)C(F)(F)C(F)(F)C(F)(F)C(F)(F)F
DTXSID6047575	UVCB_PROFLUO	FC(F)(F)C(F)(F)C(F)(F)C(F)(F)C=C
DTXSID7029904	UVCB_PROFLUO	OCCC(F)(F)C(F)(F)C(F)(F)C(F)(F)C(F)(F)C(F)(F)C(F)(F)C(F)(F)F
DTXSID70379270	UVCB_PROFLUO	CC=CC(F)(F)C(F)(F)C(F)(F)F

DTXSID	Subfamily	SMILES
DTXSID70381151	UVCB_PROFLUO	NC(=N)C(F)(F)C(F)(F)C(F)(F)C(F)(F)C(F)(F)C(F)(F)C(F)(F)F
DTXSID70400078	UVCB_PROFLUO	C[Si](C)(C)C(F)(F)C(F)(F)C(F)(F)F
DTXSID7047566	UVCB_PROFLUO	FC(F)(F)C(F)(F)C(F)(F)C(F)(F)C(F)(F)C(F)(F)I
DTXSID7060332	UVCB_PROFLUO	FC(F)(F)C(F)(F)C(F)(F)C(=O)CC(=O)C1=CC=CS1
DTXSID7061982	UVCB_PROFLUO	FC(F)(F)C1(F)C(F)(F)C(F)(F)C(F)(F)C1(F)F
DTXSID7074616	UVCB_PROFLUO	FC(F)(F)C(F)(F)C(F)(F)C(F)(F)C(F)(F)C(F)(F)C(F)(F)C(F)(F)C=C
DTXSID80310730	UVCB_PROFLUO	NC(=O)C(F)(F)C(F)(F)C(F)(F)C(F)(F)C(N)=O
DTXSID80371164	UVCB_PROFLUO	OCC(F)(OC(F)(F)C(F)(OC(F)(F)C(F)(F)C(F)(F)F)C(F)(F)F)C(F)(F)F
DTXSID80375107	UVCB_PROFLUO	OCC(F)(F)C(F)(F)C(F)(F)C(F)(F)C(F)(F)C(F)(F)C(F)(F)C(F)(F)C(F)(F)C(F)(F)C(F)(F)F
DTXSID80379721	UVCB_PROFLUO	FC(F)(COC(=O)C=C)C(F)(F)C(F)(F)C(F)(F)COC(=O)C=C
DTXSID80379781	UVCB_PROFLUO	FC(=O)C1(F)C(F)(F)C(F)(F)C(F)(F)C(F)(F)C1(F)F
DTXSID80382154	UVCB_PROFLUO	FC(F)(F)C(F)(F)C(F)(F)C(F)(F)C(F)(F)C(F)(F)C(Cl)=O
DTXSID8062101	UVCB_PROFLUO	CC(=C)C(=O)OCCC(F)(F)C(F)(F)C(F)(F)C(F)(F)C(F)(F)C(F)(F)C(F)(F)C(F)(F)F
DTXSID80881157	UVCB_PROFLUO	OCC(O)CC(F)(F)C(F)(F)C(F)(F)C(F)(F)C(F)(F)C(F)(F)C(F)(F)F
DTXSID90190949	UVCB_PROFLUO	FC(F)(I)C(F)(F)C(F)(F)C(F)(F)C(F)(F)C(F)(F)I

DTXSID	Subfamily	SMILES
DTXSID90315130	UVCB_PROFLUO	FC(F)(F)C(F)(F)C(F)(F)C(F)(F)C(F)(F)C(F)(F)C(F)(F)C(F)(F)C(F)(F)S(Cl)(=O)=O
DTXSID90371872	UVCB_PROFLUO	FC(F)(C=C)C(F)(F)C(F)(F)C(F)(F)C(F)(F)C(F)(F)C(F)(F)C(F)(F)C=C
DTXSID9038840	UVCB_PROFLUO	FC(F)(F)C(F)(F)C(F)(F)C(F)(F)C(F)(F)C(F)(F)CCOC(=O)C=C
DTXSID90558000	UVCB_PROFLUO	OP(O)(=O)OCCC(F)(F)C(F)(F)C(F)(F)C(F)(F)C(F)(F)C(F)(F)F
DTXSID9059834	UVCB_PROFLUO	FC(F)(F)C(F)(F)C(F)(F)N(C(F)(F)C(F)(F)C(F)(F)F)C(F)(F)C(F)(F)C(F)(F)F
DTXSID9059919	UVCB_PROFLUO	FC(F)(F)C(F)(F)C(F)(F)C(F)(F)C(F)(F)C(F)(F)C(F)(F)Br
DTXSID9061881	UVCB_PROFLUO	FC(F)(F)C(F)(F)C(F)(F)C(F)(F)C(F)(F)CCI
DTXSID90880156	UVCB_PROFLUO	FC(F)(F)C(F)(F)C(F)(F)C(F)(F)C(F)(F)C(F)(F)C(F)(F)CCI

S5. Butina-cutoff split stability

Per-fold compound counts of the 5-fold Tanimoto-Butina partition at similarity cutoffs 0.5–0.8, showing the partition is insensitive to the exact cutoff in this range (Table S5).

Table S5: Stability of the 5-fold Tanimoto-Butina partition of the 209 PFAS to the similarity cutoff: per-fold compound counts at cutoffs 0.5–0.8. The partition is essentially unchanged across this range, so the reported splits are not fragile to the exact cutoff.

Cutoff	Fold 1	Fold 2	Fold 3	Fold 4	Fold 5	mean
0.5	42	42	42	42	41	41.8

Cutoff	Fold 1	Fold 2	Fold 3	Fold 4	Fold 5	mean
0.6	42	42	42	42	41	41.8
0.7	42	42	42	42	41	41.8
0.8	42	42	42	42	41	41.8

S6. Conformal calibration detail

Calibration protocols. All conformal results use predictions from the off-the-shelf multi-task model; the conformal layer performs no model refitting and only estimates per-class score quantiles. We report three protocols. The *recycled-fold* protocol calibrates on folds 0–2 and tests on folds 3–4; because the fold-3/4 models were trained with the fold-0–2 compounds, calibration and test compounds are not strictly exchangeable, so it is approximate, but its larger calibration set makes the Mondrian quantiles genuinely data-driven, and we treat it as the substantive result. The *true-holdout* protocol is exchangeability-respecting: for each fold k, the fold-k model (trained on the other four folds) scores the held-out fold-k compounds, which are split 50/50 into a calibration set ($n_{\text{cal}} \approx 20$) and a test set ($n_{\text{test}} \approx 21$), both held out of the scoring model; we repeat the random split 200 times per fold and report the mean and SD over the 1,000 evaluations. The *pooled* protocol pools the out-of-fold predictions of all five fold-models into one set (209 compound-rows, each compound scored by the model that held its fold out) and takes 200 random 50/50 calibration/test splits, giving a $\sim$4× larger per-endpoint calibration set ($\sim$83 compounds median) at the cost of mixing five fold-models, so its exchangeability is also approximate.

Quantile estimation and fallback. Per endpoint, the class-conditional quantile $\hat{q}_y$ is the $\lceil (n_y + 1)(1 - \alpha) \rceil / n_y$ empirical quantile of the class-y calibration non-conformity scores, with a conservative fallback ($\hat{q}_y = 0.95$) when a class has fewer than 5 calibration compounds. At $n_{\text{cal}} \approx 20$ this fallback fires for $\sim$74% of endpoints, so the true-holdout figures are fallback-

influenced; pooling drops the fallback rate to 1%. Empty prediction sets are resolved to the arg-max class, which slightly relaxes the exact split-conformal guarantee. Given the small calibration sizes, the conformal analysis is presented as an exploratory calibration protocol rather than a coverage-guaranteed result.

Per-fold and pooled conformal-coverage detail supporting main-text Table 3 are given in Table S6.

Table S6: Conformal calibration detail (companion to main-text Table 3). Top: repeated true-holdout protocol, per-fold mean marginal coverage over 200 splits each. Bottom: pooled-holdout sweep over the calibration fraction.

Repeated true-holdout (per-fold marginal coverage, 200 splits/fold)						
Fold	1	2	3	4	5	overall
Marginal	0.921	0.921	0.926	0.936	0.923	0.925
Pooled-holdout sweep (calibration fraction)						
Cal. frac	n_cal	marginal	pos-class	neg-class	fallback%	singleton%
0.5	104	0.923	0.934	0.919	1	30
0.6	125	0.923	0.938	0.919	1	29
0.7	146	0.923	0.946	0.916	0	27

S7. Threshold-dependent metrics

The main text reports threshold-free metrics (AUROC, AUPRC). Because the active class is rare (mean active rate 0.208 across the 156 endpoints, range 0.044–0.550), Table S7 adds F1, Matthews correlation coefficient and balanced accuracy at two explicit operating points: the cut that maximises F1 on the *training* fold only, and a fixed 0.5 cut. No held-out information is used to select either.

Table S7: Threshold-dependent metrics under class imbalance, over all evaluable (endpoint, fold) pairs of the 5-fold Tanimoto-Butina split. The operating point is either the cut maximising F1 on the training fold only, or a fixed 0.5.

Model	Threshold rule	F1	MCC	Balanced acc.	Precision	Recall
MolFormer multi-task	fixed 0.5	0.256 ± 0.247	0.202 ± 0.239	0.583 ± 0.106	0.398	0.231
MolFormer multi-task	train-fold F1-optimal	0.432 ± 0.229	0.292 ± 0.258	0.658 ± 0.135	0.412	0.599
Morgan-FP + XGBoost	fixed 0.5	0.236 ± 0.204	0.120 ± 0.204	0.553 ± 0.092	0.313	0.225
Morgan-FP + XGBoost	train-fold F1-optimal	0.202 ± 0.206	0.110 ± 0.202	0.545 ± 0.084	0.303	0.181

The training-fold F1-optimal cut transfers worse than a fixed 0.5 cut for the XGBoost baseline. This is an artefact of `scale_pos_weight` rebalancing, which drives the training-fold probabilities to a near-degenerate optimum; the fixed-0.5 rows are the like-for-like comparison.

S8. Structured label missingness

ToxCast screening of PFAS is organised around the EPA PFAS inventory, so the label matrix is not missing at random. Table S8 reports endpoints tested per PFAS by subfamily and by cross-validation fold, together with the tests showing that the missingness pattern is aligned with chemical clustering but not with the cross-validation partition.

Table S8: Structured label missingness: endpoints tested per PFAS, by subfamily and by cross-validation fold. "Active rate" is the fraction of labelled (compound, endpoint) pairs that are active.

Subfamily	n	mean tested	median	min	max	active rate
FOSA/FOSE	8	136.6	156	1	156	0.567
FTOH	15	115.5	156	1	156	0.173
OTHER	49	93.2	155	1	156	0.175
PFCA	25	143.8	156	7	156	0.206
PFECA	7	155.9	156	155	156	0.216
PFESA	1	155.0	155	155	155	0.097
PFSA	11	143.7	156	92	156	0.187

Kruskal–Wallis across subfamilies $H = 22.3$, $p = 2.2e-03$, $\eta^2 = 0.098$; across the 5 cluster-out folds $H = 7.41$, $p = 0.116$, $\eta^2 = 0.026$. Jaccard similarity between two compounds' label masks is 0.930 within a Butina cluster vs. 0.644 between (permutation $p < 5 \times 10^{-4}$), but only 0.652 vs. 0.648 within vs. between CV fold: the missingness is structured by chemistry but not by the partition.

Subfamily	n	mean tested	median	min	max	active rate
UVCB/PROFLUO	93	129.2	156	1	156	0.196
CV fold 1	42	121.7	156	–	–	–
CV fold 2	42	131.0	156	–	–	–
CV fold 3	42	114.8	156	–	–	–
CV fold 4	42	137.7	156	–	–	–
CV fold 5	41	112.4	156	–	–	–

Kruskal–Wallis across subfamilies $H = 22.3$, $p = 2.2e-03$, $\eta^2 = 0.098$; across the 5 cluster-out folds $H = 7.41$, $p = 0.116$, $\eta^2 = 0.026$. Jaccard similarity between two compounds' label masks is 0.930 within a Butina cluster vs. 0.644 between (permutation $p < 5 \times 10^{-4}$), but only 0.652 vs. 0.648 within vs. between CV fold: the missingness is structured by chemistry but not by the partition.

S9. Full representation sweep

Table S9 gives the complete eight-representation sweep underlying main-text Table 2, adding macro AUPRC for every cell and the fingerprint-collision statistics that motivate it.

Table S9: Full representation sweep on the same 5-fold Tanimoto-Butina split as the main comparison, with AUPRC included. Reference: fine-tuned MolFormer-XL with multi-task heads reaches macro AUROC 0.764 ± 0.100, macro AUPRC 0.530 ± 0.170.

Representation	dim	XGB AUROC	XGB AUPRC	MLP-MT AUROC	MLP-MT AUPRC
1D composition (12 features)	12	0.637 ± 0.052	0.373 ± 0.064	0.610 ± 0.131	0.385 ± 0.110
Morgan bits r=2 (submitted baseline)	672	0.589 ± 0.029	0.376 ± 0.058	0.561 ± 0.074	0.341 ± 0.060
Morgan bits r=3	961	0.591 ± 0.059	0.405 ± 0.087	0.575 ± 0.070	0.373 ± 0.063
Morgan counts r=2	673	0.595 ± 0.096	0.388 ± 0.102	0.638 ± 0.084	0.414 ± 0.084
MACCS keys	134	0.544 ± 0.055	0.326 ± 0.041	0.554 ± 0.084	0.334 ± 0.071
RDKit 2D descriptors	172	0.688 ± 0.074	0.459 ± 0.131	0.658 ± 0.086	0.418 ± 0.105
RDKit 2D + Morgan counts	845	0.687 ± 0.073	0.458 ± 0.130	0.683 ± 0.069	0.430 ± 0.091

3D conformer shape + AUTOCORR3D	91	0.603 ± 0.045	0.341 ± 0.080	0.580 ± 0.079	0.330 ± 0.080

Binary Morgan r=2 maps 54 of 209 compounds onto duplicate vectors; of the 8,995 label pairs inside those groups, 3,316 (36.9%) disagree, across all 156 endpoints. Count-based Morgan, the RDKit 2D block and the 3D block each resolve all 209 compounds uniquely.

S10. Applicability domain

Table S10 reports accuracy by quartile of similarity to the training fold, and how much of the untested PFASSTRUCT library lies inside the domain at fixed similarity thresholds.

Table S10: Applicability domain. Upper block: held-out compounds binned by quartile of similarity to their training fold (mean Tanimoto to the 5 nearest training compounds), with macro AUROC recomputed inside each bin. Lower block: how many of the 20,793 untested PFASSTRUCT compounds fall inside the domain at fixed thresholds.

Bin	compounds	similarity range	endpoint-folds	macro AUROC
Q1 (least similar to training)	53	0.10–0.35	451	0.737
Q2	53	0.35–0.46	635	0.724
Q3	52	0.46–0.54	511	0.730
Q4 (most similar to training)	51	0.54–0.72	629	0.759
Library within mean-top-5 Tanimoto 0.3	9,758	≥ 0.3	–	–
Library within mean-top-5 Tanimoto 0.4	5,455	≥ 0.4	–	–
Library within mean-top-5 Tanimoto 0.5	2,543	≥ 0.5	–	–
Library within mean-top-5 Tanimoto 0.6	1,043	≥ 0.6	–	–
Library within mean-top-5 Tanimoto 0.7	461	≥ 0.7	–	–

Accuracy is flat across the range the benchmark spans, but most of the untested PFAS library lies further from the training set than any evaluated compound.

S11. Repeated cross-validation

Table S11 repeats the entire 5-fold protocol under five independent Butina partitions, each retraining the headline model from scratch, to separate partition-specific variation from the reported effect.

Table S11: Repeated 5-fold Tanimoto-Butina cross-validation. Each seed regenerates the cluster partition and retrains the headline model from scratch on all five folds.

Split seed	fold sizes	per-fold macro AUROC	mean ± SD
42	42, 42, 42, 42, 41	0.835, 0.762, 0.749, 0.590, 0.883	0.764 ± 0.100
7	42, 42, 42, 42, 41	0.799, 0.719, 0.811, 0.581, 0.782	0.738 ± 0.085
123	42, 42, 42, 42, 41	0.754, 0.778, 0.830, 0.709, 0.783	0.771 ± 0.040
2024	42, 42, 42, 42, 41	0.842, 0.823, 0.597, 0.655, 0.754	0.734 ± 0.095
31337	42, 42, 42, 42, 41	0.869, 0.812, 0.623, 0.646, 0.789	0.748 ± 0.096

Across the 5 independent partitions the mean is 0.751 ± 0.014 (range 0.734–0.771). Seed 42 is the partition used throughout the main text. The across-partition spread is roughly seven times smaller than the within-partition fold-to-fold spread.

S12. Catalogue of the 156 ToxCast endpoints

Table S12 lists every endpoint used in this study with its invitroDB v4.3 assay metadata (intended target family, assay function type, detection technology) and its PFAS counts, and Table S13 rolls the panel up by target family. The surviving panel is dominated by cell-based phenotypic readouts rather than by transcription-factor assays: the latter are removed by the ≥10-active filter because essentially no tested PFAS is active in them.

Table S12: Catalogue of the 156 ToxCast endpoints used in this study. "PFAS tested" and "actives" are counts over the 209-compound evaluation union; endpoints were selected by requiring at least 100 PFAS tested and at least 10 actives. Assay metadata are from invitroDB v4.3.

aeid	Assay endpoint	Target family	Function	Detection	PFAS tested	Actives	Active
146	BSK_3C_Eselectin	cell adhesion molecules	signaling	fluorescence	168	32	0.19

aeid	Assay endpoint	Target family	Function	Detection	PFAS tested	Actives	Active
148	BSK_3C_HLADR	cell adhesion molecules	signaling	fluorescence	168	35	0.20
150	BSK_3C_ICAM1	cell adhesion molecules	signaling	fluorescence	168	21	0.12
152	BSK_3C_IL8	cytokine	signaling	fluorescence	168	44	0.26
154	BSK_3C_MCP1	cytokine	signaling	fluorescence	168	38	0.22
156	BSK_3C_MIG	cytokine	signaling	fluorescence	168	19	0.11
158	BSK_3C_Proliferation	cell cycle	viability	absorbance	168	65	0.38
160	BSK_3C_SRB	cell morphology	signaling	absorbance	168	45	0.26
162	BSK_3C_Thrombomodulin	gpcr	signaling	fluorescence	168	40	0.23
164	BSK_3C_TissueFactor	cytokine	signaling	fluorescence	168	32	0.19
166	BSK_3C_uPAR	cytokine	signaling	fluorescence	168	34	0.20
168	BSK_3C_VCAM1	cell adhesion molecules	signaling	fluorescence	168	28	0.16
172	BSK_4H_Eotaxin3	cytokine	signaling	fluorescence	168	55	0.32
174	BSK_4H_MCP1	cytokine	signaling	fluorescence	168	27	0.16
176	BSK_4H_Pselectin	cell adhesion molecules	signaling	fluorescence	168	44	0.26
178	BSK_4H_SRB	cell morphology	signaling	absorbance	168	35	0.20
180	BSK_4H_uPAR	cytokine	signaling	fluorescence	168	38	0.22
182	BSK_4H_VCAM1	cell adhesion molecules	signaling	fluorescence	168	41	0.24
184	BSK_4H_VEGFRII	kinase	signaling	fluorescence	168	37	0.22
186	BSK_BE3C_HLADR	cell adhesion molecules	signaling	fluorescence	168	43	0.25
188	BSK_BE3C_IL1a	cytokine	signaling	fluorescence	168	37	0.22
190	BSK_BE3C_IP10	cytokine	signaling	fluorescence	168	46	0.27
192	BSK_BE3C_MIG	cytokine	signaling	fluorescence	168	26	0.15
194	BSK_BE3C_MMP1	protease	signaling	fluorescence	168	76	0.45
196	BSK_BE3C_PAI1	cytokine	signaling	fluorescence	168	36	0.21
198	BSK_BE3C_SRB	cell morphology	signaling	absorbance	168	23	0.13
202	BSK_BE3C_tPA	protease	signaling	fluorescence	168	39	0.23
204	BSK_BE3C_uPA	protease	signaling	fluorescence	168	28	0.16
206	BSK_BE3C_uPAR	cytokine	signaling	fluorescence	168	29	0.17
208	BSK_CASM3C_HLADR	cell adhesion molecules	signaling	fluorescence	168	28	0.16
210	BSK_CASM3C_IL6	cytokine	signaling	fluorescence	168	19	0.11
212	BSK_CASM3C_IL8	cytokine	signaling	fluorescence	168	19	0.11
214	BSK_CASM3C_LDLR	misc protein	signaling	fluorescence	168	30	0.17

aeid	Assay endpoint	Target family	Function	Detection	PFAS tested	Actives	Active
216	BSK_CASM3C_MCP1	cytokine	signaling	fluorescence	168	16	0.09
218	BSK_CASM3C_MCSF	cytokine	signaling	fluorescence	168	32	0.19
220	BSK_CASM3C_MIG	cytokine	signaling	fluorescence	168	39	0.23
222	BSK_CASM3C_Proliferation	cell cycle	viability	absorbance	168	46	0.27
224	BSK_CASM3C_SAA	cell adhesion molecules	signaling	fluorescence	168	35	0.20
226	BSK_CASM3C_SRB	cell morphology	signaling	absorbance	168	17	0.10
228	BSK_CASM3C_Thrombomodulin	gpcr	signaling	fluorescence	168	25	0.14
230	BSK_CASM3C_TissueFactor	cytokine	signaling	fluorescence	168	21	0.12
232	BSK_CASM3C_uPAR	cytokine	signaling	fluorescence	168	15	0.08
234	BSK_CASM3C_VCAM1	cell adhesion molecules	signaling	fluorescence	168	44	0.26
236	BSK_hDFCGF_CollagenIII	cell adhesion molecules	signaling	fluorescence	168	48	0.28
238	BSK_hDFCGF_EGFR	kinase	signaling	fluorescence	168	38	0.22
240	BSK_hDFCGF_IL8	cytokine	signaling	fluorescence	168	21	0.12
242	BSK_hDFCGF_IP10	cytokine	signaling	fluorescence	168	56	0.33
244	BSK_hDFCGF_MCSF	cytokine	signaling	fluorescence	168	49	0.29
246	BSK_hDFCGF_MIG	cytokine	signaling	fluorescence	168	54	0.32
248	BSK_hDFCGF_MMP1	protease	signaling	fluorescence	168	35	0.20
250	BSK_hDFCGF_PAI1	cytokine	signaling	fluorescence	168	35	0.20
252	BSK_hDFCGF_Proliferation	cell cycle	viability	absorbance	168	59	0.35
254	BSK_hDFCGF_SRB	cell morphology	signaling	absorbance	168	68	0.40
256	BSK_hDFCGF_TIMP1	protease inhibitor	signaling	fluorescence	168	33	0.19
258	BSK_hDFCGF_VCAM1	cell adhesion molecules	signaling	fluorescence	168	55	0.32
260	BSK_KF3CT_ICAM1	cell adhesion molecules	signaling	fluorescence	168	25	0.14
262	BSK_KF3CT_IL1a	cytokine	signaling	fluorescence	168	32	0.19
264	BSK_KF3CT_IP10	cytokine	signaling	fluorescence	168	31	0.18
266	BSK_KF3CT_MCP1	cytokine	signaling	fluorescence	168	30	0.17
268	BSK_KF3CT_MMP9	protease	signaling	fluorescence	168	45	0.26
270	BSK_KF3CT_SRB	cell morphology	signaling	absorbance	168	25	0.14
274	BSK_KF3CT_TIMP2	protease inhibitor	signaling	fluorescence	168	33	0.19
276	BSK_KF3CT_uPA	protease	signaling	fluorescence	168	23	0.13
278	BSK_LPS_CD40	cytokine	signaling	fluorescence	168	47	0.28
280	BSK_LPS_Eselectin	cell adhesion molecules	signaling	fluorescence	168	29	0.17
2809	BSK_BT_Bcell_Proliferation	cell cycle	viability	absorbance	160	48	0.30

aeid	Assay endpoint	Target family	Function	Detection	PFAS tested	Actives	Active
2811	BSK_BT_PBMCCytotoxicity	cell cycle	viability	fluorescence	160	39	0.24
2813	BSK_BT_sIgG	immunoglobulin	signaling	fluorescence	160	33	0.20
2815	BSK_BT_xIL17A	cytokine	signaling	fluorescence	160	30	0.18
2817	BSK_BT_xIL17F	cytokine	signaling	fluorescence	160	46	0.28
2819	BSK_BT_xIL2	cytokine	signaling	fluorescence	160	28	0.17
282	BSK_LPS_IL1a	cytokine	signaling	fluorescence	168	37	0.22
2821	BSK_BT_xIL6	cytokine	signaling	fluorescence	160	26	0.16
2823	BSK_BT_xTNFa	cytokine	signaling	fluorescence	160	88	0.55
2825	BSK_MyoF_ACTA1	cell adhesion molecules	signaling	fluorescence	160	37	0.23
2827	BSK_MyoF_bFGF	growth factor	signaling	fluorescence	160	16	0.10
2829	BSK_MyoF_VCAM1	cell adhesion molecules	signaling	fluorescence	160	32	0.20
2831	BSK_MyoF_CollagenI	cell adhesion molecules	signaling	fluorescence	160	30	0.18
2833	BSK_MyoF_CollagenIII	cell adhesion molecules	signaling	fluorescence	160	30	0.18
2835	BSK_MyoF_CollagenIV	cell adhesion molecules	signaling	fluorescence	160	31	0.19
2837	BSK_MyoF_IL8	cytokine	signaling	fluorescence	160	22	0.13
2839	BSK_MyoF_Decorin	cell adhesion molecules	signaling	fluorescence	160	31	0.19
284	BSK_LPS_IL8	cytokine	signaling	fluorescence	168	59	0.35
2841	BSK_MyoF_MMP1	protease	signaling	fluorescence	160	24	0.15
2843	BSK_MyoF_PAI1	cytokine	signaling	fluorescence	160	23	0.14
2845	BSK_MyoF_SRB	cell morphology	signaling	absorbance	160	27	0.16
2847	BSK_MyoF_TIMP1	protease	signaling	fluorescence	160	25	0.15
2849	BSK_BF4T_MCP1	cytokine	signaling	fluorescence	160	55	0.34
2851	BSK_BF4T_Eotaxin3	cytokine	signaling	fluorescence	160	40	0.25
2853	BSK_BF4T_VCAM1	cell adhesion molecules	signaling	fluorescence	160	32	0.20
2855	BSK_BF4T_ICAM1	cell adhesion molecules	signaling	fluorescence	160	22	0.13
2857	BSK_BF4T_CD90	cytokine	signaling	fluorescence	160	13	0.08
2859	BSK_BF4T_IL8	cytokine	signaling	fluorescence	160	39	0.24
286	BSK_LPS_MCP1	cytokine	signaling	fluorescence	168	34	0.20
2861	BSK_BF4T_IL1a	cytokine	signaling	fluorescence	160	21	0.13
2863	BSK_BF4T_Keratin818	cell adhesion molecules	signaling	fluorescence	160	22	0.13

aeid	Assay endpoint	Target family	Function	Detection	PFAS tested	Actives	Active
2865	BSK_BF4T_MMP1	protease	signaling	fluorescence	160	17	0.10
2867	BSK_BF4T_MMP3	protease	signaling	fluorescence	160	43	0.26
2869	BSK_BF4T_MMP9	protease	signaling	fluorescence	160	30	0.18
2871	BSK_BF4T_PAI1	cytokine	signaling	fluorescence	160	23	0.14
2873	BSK_BF4T_SRB	cell morphology	signaling	absorbance	160	18	0.11
2875	BSK_BF4T_tPA	protease	signaling	fluorescence	160	39	0.24
2877	BSK_BF4T_uPA	protease	signaling	fluorescence	160	20	0.12
2879	BSK_BE3C_ICAM1	cell adhesion molecules	signaling	fluorescence	160	23	0.14
288	BSK_LPS_MCSF	cytokine	signaling	fluorescence	168	36	0.21
2881	BSK_BE3C_ITAC	cytokine	signaling	fluorescence	160	35	0.21
2883	BSK_BE3C_IL8	cytokine	signaling	fluorescence	160	48	0.30
2885	BSK_BE3C_EGFR	kinase	signaling	fluorescence	160	24	0.15
2887	BSK_BE3C_Keratin818	cell adhesion molecules	signaling	fluorescence	160	22	0.13
2889	BSK_BE3C_MMP9	protease	signaling	fluorescence	160	41	0.25
2891	BSK_CASM3C_PAI1	cytokine	signaling	fluorescence	160	7	0.04
2893	BSK_hDFCGF_MCP1	cytokine	signaling	fluorescence	160	45	0.28
2895	BSK_hDFCGF_ICAM1	cell adhesion molecules	signaling	fluorescence	160	34	0.21
2897	BSK_hDFCGF_CollagenI	cell adhesion molecules	signaling	fluorescence	160	23	0.14
2899	BSK_hDFCGF_ITAC	cytokine	signaling	fluorescence	160	66	0.41
290	BSK_LPS_PGE2	gpcr	signaling	fluorescence	168	34	0.20
2901	BSK_hDFCGF_TIMP2	protease	signaling	fluorescence	160	51	0.31
2903	BSK_KF3CT_IL8	cytokine	signaling	fluorescence	160	34	0.21
2905	BSK_KF3CT_MIG	cytokine	signaling	fluorescence	160	23	0.14
2907	BSK_KF3CT_PAI1	cytokine	signaling	fluorescence	160	37	0.23
2909	BSK_IMphg_MCP1	cytokine	signaling	fluorescence	160	32	0.20
2911	BSK_IMphg_MIP1a	cytokine	signaling	fluorescence	160	33	0.20
2913	BSK_IMphg_VCAM1	cell adhesion molecules	signaling	fluorescence	160	33	0.20
2915	BSK_IMphg_CD40	cytokine	signaling	fluorescence	160	50	0.31
2917	BSK_IMphg_ESelectin	cell adhesion molecules	signaling	fluorescence	160	38	0.23
2919	BSK_IMphg_CD69	cytokine	signaling	fluorescence	160	41	0.25
292	BSK_LPS_SRB	cell morphology	signaling	absorbance	168	30	0.17
2921	BSK_IMphg_IL8	cytokine	signaling	fluorescence	160	49	0.30
2923	BSK_IMphg_IL1a	cytokine	signaling	fluorescence	160	26	0.16

aeid	Assay endpoint	Target family	Function	Detection	PFAS tested	Actives	Active
2925	BSK_IMphg_MCSF	cytokine	signaling	fluorescence	160	19	0.11
2927	BSK_IMphg_IL10	cytokine	signaling	fluorescence	160	58	0.36
2929	BSK_IMphg_SRB	cell morphology	signaling	absorbance	160	21	0.13
2931	BSK_IMphg_SRB.Mphg	cell morphology	signaling	absorbance	160	55	0.34
2933	BSK_LPS_Thrombomodulin	gpcr	signaling	fluorescence	160	42	0.26
2935	BSK_LPS_CD69	cytokine	signaling	fluorescence	160	47	0.29
294	BSK_LPS_TissueFactor	cytokine	signaling	fluorescence	168	38	0.22
296	BSK_LPS_TNFa	cytokine	signaling	fluorescence	168	35	0.20
298	BSK_LPS_VCAM1	cell adhesion molecules	signaling	fluorescence	168	39	0.23
300	BSK_SAg_CD38	cytokine	signaling	fluorescence	168	41	0.24
302	BSK_SAg_CD40	cytokine	signaling	fluorescence	168	45	0.26
304	BSK_SAg_CD69	cytokine	signaling	fluorescence	168	41	0.24
306	BSK_SAg_Eselectin	cell adhesion molecules	signaling	fluorescence	168	35	0.20
308	BSK_SAg_IL8	cytokine	signaling	fluorescence	168	40	0.23
310	BSK_SAg_MCP1	cytokine	signaling	fluorescence	168	33	0.19
312	BSK_SAg_MIG	cytokine	signaling	fluorescence	168	18	0.10
314	BSK_SAg_PBMCCytotoxicity	cell cycle	viability	fluorescence	168	42	0.25
316	BSK_SAg_Proliferation	cell cycle	viability	absorbance	168	45	0.26
318	BSK_SAg_SRB	cell morphology	signaling	absorbance	168	34	0.20
3211	CCTE_Padilla_ZF_Score.Living	zebrafish development	developmental	fluorescence microscopy	182	24	0.13
3213	CCTE_Padilla_ZF_Score.General	zebrafish development	developmental	fluorescence microscopy	182	15	0.08
3214	CCTE_Padilla_ZF_Score.Swim_bladder	zebrafish development	developmental	fluorescence microscopy	182	26	0.14
3215	CCTE_Padilla_ZF_Score.Craniofacial	zebrafish development	developmental	fluorescence microscopy	182	15	0.08
3216	CCTE_Padilla_ZF_Score.Edema	zebrafish development	developmental	fluorescence microscopy	182	17	0.09
3219	CCTE_Padilla_ZF_Score.Position	zebrafish development	developmental	fluorescence microscopy	182	10	0.05
3222	CCTE_Padilla_ZF_Score.Any	zebrafish development	developmental	fluorescence microscopy	182	26	0.14
75	ATG_ERE_CIS	nuclear receptor	reporter gene	fluorescence	198	9	0.04

Table S13: The 156 endpoints rolled up by intended target family (invitroDB v4.3). Counts are over the 209-compound evaluation union.

Intended target family	endpoints	median PFAS tested	median actives	mean active rate
cytokine	71	168	35	0.217
cell adhesion molecules	31	168	32	0.200
protease	15	160	35	0.218
cell morphology	12	168	28	0.200
cell cycle	7	168	46	0.296
zebrafish development	7	182	17	0.104
gpcr	4	168	37	0.213
kinase	3	168	37	0.199
protease inhibitor	2	168	33	0.196
misc protein	1	168	30	0.179
immunoglobulin	1	160	33	0.206
growth factor	1	160	16	0.100
nuclear receptor	1	198	9	0.045
All endpoints	156	168	34	0.208
